# Alternative splicing in normal and pathological human placentas is correlated to genetic variants

**DOI:** 10.1007/s00439-020-02248-x

**Published:** 2021-01-12

**Authors:** Camino S. M. Ruano, Clara Apicella, Sébastien Jacques, Géraldine Gascoin, Cassandra Gaspar, Francisco Miralles, Céline Méhats, Daniel Vaiman

**Affiliations:** 1 Université de Paris, Institut Cochin, Inserm U1016, CNRS, 24 rue du Faubourg St Jacques, 75014 Paris, France; 2 Unité Mixte de Recherche MITOVASC, Équipe Mitolab, CNRS 6015, INSERM U1083, Université d’Angers, Angers, France; 3 Réanimation et Médecine Néonatales, Centre Hospitalier Universitaire, Angers, France; 4 Sorbonne Université, Inserm, UMS Production et Analyse des Données en Sciences de la vie et en Santé, PASS, Plateforme Post-génomique de la Pitié-Salpêtrière, P3S, 75013 Paris, France

## Abstract

Two major obstetric diseases, preeclampsia (PE), a pregnancy-induced endothelial dysfunction leading to hypertension and proteinuria, and intra-uterine growth-restriction (IUGR), a failure of the fetus to acquire its normal growth, are generally triggered by placental dysfunction. Many studies have evaluated gene expression deregulations in these diseases, but none has tackled systematically the role of alternative splicing. In the present study, we show that alternative splicing is an essential feature of placental diseases, affecting 1060 and 1409 genes in PE vs controls and IUGR vs controls, respectively, many of those involved in placental function. While in IUGR placentas, alternative splicing affects genes specifically related to pregnancy, in preeclamptic placentas, it impacts a mix of genes related to pregnancy and brain diseases. Also, alternative splicing variations can be detected at the individual level as sharp splicing differences between different placentas. We correlate these variations with genetic variants to define splicing Quantitative Trait Loci (sQTL) in the subset of the 48 genes the most strongly alternatively spliced in placental diseases. We show that alternative splicing is at least partly piloted by genetic variants located either in cis (52 QTL identified) or in trans (52 QTL identified). In particular, we found four chromosomal regions that impact the splicing of genes in the placenta. The present work provides a new vision of placental gene expression regulation that warrants further studies.

**Supplementary Information:**

The online version contains supplementary material available at 10.1007/s00439-020-02248-x.

## Introduction

Preeclampsia (PE) is a major disease of pregnancy, marked by hypertension, proteinuria, and a generalized endothelial maternal disorder (Sibai et al. 2005; Rana et al. 2019). PE induces 70,000 maternal deaths and ~ 1,000,000 fetal deaths worldwide (1.5–10% of pregnancies according to the country, with a high prevalence in sub-Saharan Africa). IUGR, a failure of the fetus to reach its normal growth potential, affects up to 10% of the fetuses, and is a major cause of the birth of small babies, that are at risk for acute diseases of the retina (Gupta et al. 2008), the lung (Arroyas et al. 2020; Fandino et al. 2019; Zana-Taieb et al. 2015), the heart (Crispi et al. 2008) (in the most severe cases) and long term increased risk of cardiovascular diseases (less severe cases) (Jansson and Powell 2007; Savage et al. 2020). These two gestational pathologies are strongly connected since one third of the preeclampsia cases results in IUGR. PE and IUGR are accompanied by epigenetic alterations at the placental level and have been strongly associated with an imbalance of the oxidative and nitrosative stresses (Myatt 2010; Aouache and Biquard 2018; Doridot et al. 2014). One of the earliest markers of preeclampsia is an excessive increase of the soluble form of the VEGF receptor, sFLT1, in the maternal plasma (Souders et al. 2015). This form is generated by an alternative exon splicing event that will generate a protein encoded by the first 13–15 exons of the FLT1 gene (Szalai et al. 2015).

This soluble receptor is classically thought to perform as a trap for the VEGF and PGF angiogenesis factors (Phipps et al. 2019). Thus, it is classically assumed that an exon-splicing event of FLT1 is a major causative factor in the onset of PE. The idea that alternative splicing mechanisms are more general in the pathophysiology of pregnancy, including IUGR, has not been systematically analyzed so far.

Exon splicing events on a global scale are currently theoretically measurable by two technical approaches: exon-specific microarrays and RNA sequencing. Nevertheless, splicing events are rarely mentioned in the RNA-seq experiments carried out in normal and pathological placental samples, with a recent exception (Majewska et al. 2019). To address alternative splicing, RNA-seq need very high depth of reads (60–100X), and paired-end reads. These requirements are seldom achieved and studies using RNA-seq approaches on preeclamptic placental or decidual samples do not dive into details on splicing profile (Tong et al. 2018; Sober et al. 2015). This is also true for other defective pregnancy outcomes, such as recurrent miscarriage (Sober et al. 2016). Notwithstanding, genes involved in splicing and mRNA processing are highly expressed both in normal and pathological placentas (Aouache and Biquard 2018).

In the present study, we decided to thoroughly analyze alternative splicing events in the placenta in the context of pathological pregnancies. We profiled placental RNA levels from controls, preeclamptic patients and IUGR patients at the exon level using exon-microarrays. In parallel, the same patients and controls were genotyped using SNP-microarray technology. This design allowed us to discover extensive alterations of splicing throughout the placental genome in pathological situations. In addition, we identified genetic variants that are associated to splicing modifications, located in *cis* and *trans*, with or without connection with the disease status. These splicing Quantitative Trait Loci (sQTLs) may thus reflect individual-specific regulations of placental functions.

## Results

### A genomic search for alternative splicing in the human placenta

#### Placental alternative splicing is a prominent feature of gestational diseases

To determine whether there are differences in mature transcripts for a given gene between healthy and diseased placentas, gene expression at the exon level was measured in placentas from two distinct cohorts of patients: a cohort of PE-affected women and matched controls (Institut Cochin-PE cohort, *n* = 7 and 9, respectively) and another of IUGR-affected women and matched controls (Angers Hospital-IUGR cohort, *n* = 13 and 8, respectively). Patient characteristics are presented in Supplementary Table S1. As expected, both gestational age and birth weight are significantly different between disease and control groups. This difference is a recurrent and expected feature of transcriptome analysis comparing normal and pathological placentas. In this case, since most of the samples (including the pathological ones) are from third-trimester placentas, the effects linked to placental age are probably meek compared to the ones associated with the disease per se. To more thoroughly ascertain this, we proceeded to identify and estimate surrogate variables for known and unknown sources of variation in our dataset using the SVA package (Leek et al. 2012). In the IUGR vs CTRL comparison, 6.5% of the probes differentially expressed according to disease, were also associated to gestational age, 12.5% to birth weight, and 10.9% to sex. In the PE vs CTRL comparison, 7.5% of the genes significantly changed due to PE effects were significantly associated to gestational age, 6.8% to birthweight and 10% to the sex of the baby. Considering these data, we assumed that the gene expression and splicing alterations detected are strongly influenced by the disease status. These data are summarized as Supplemental Table S2a, S2b and S2c for the comparison between CTLs on the one hand, vs IUGRs, all PEs, and isolated PEs, respectively.

The comparison of gene expression at the exon level was carried out using Affymetrix ClariomD microarray, an attractive solution for tackling the complex question of alternative splicing regulation, compared to other more computation-intensive approaches depending upon RNA-seq. Analyses through the Affymetrix Transcriptome Analysis Console software lead to the definition of a splicing index associated with a relative* p* value for each gene and each comparison (Gardina et al. 2006), allowing to rank the genes from the most differentially spliced, to the least. The complete dataset is available with the accession number E-MTAB-9416 (EMBL-EBI ArrayExpress).

**Fig. 1:**
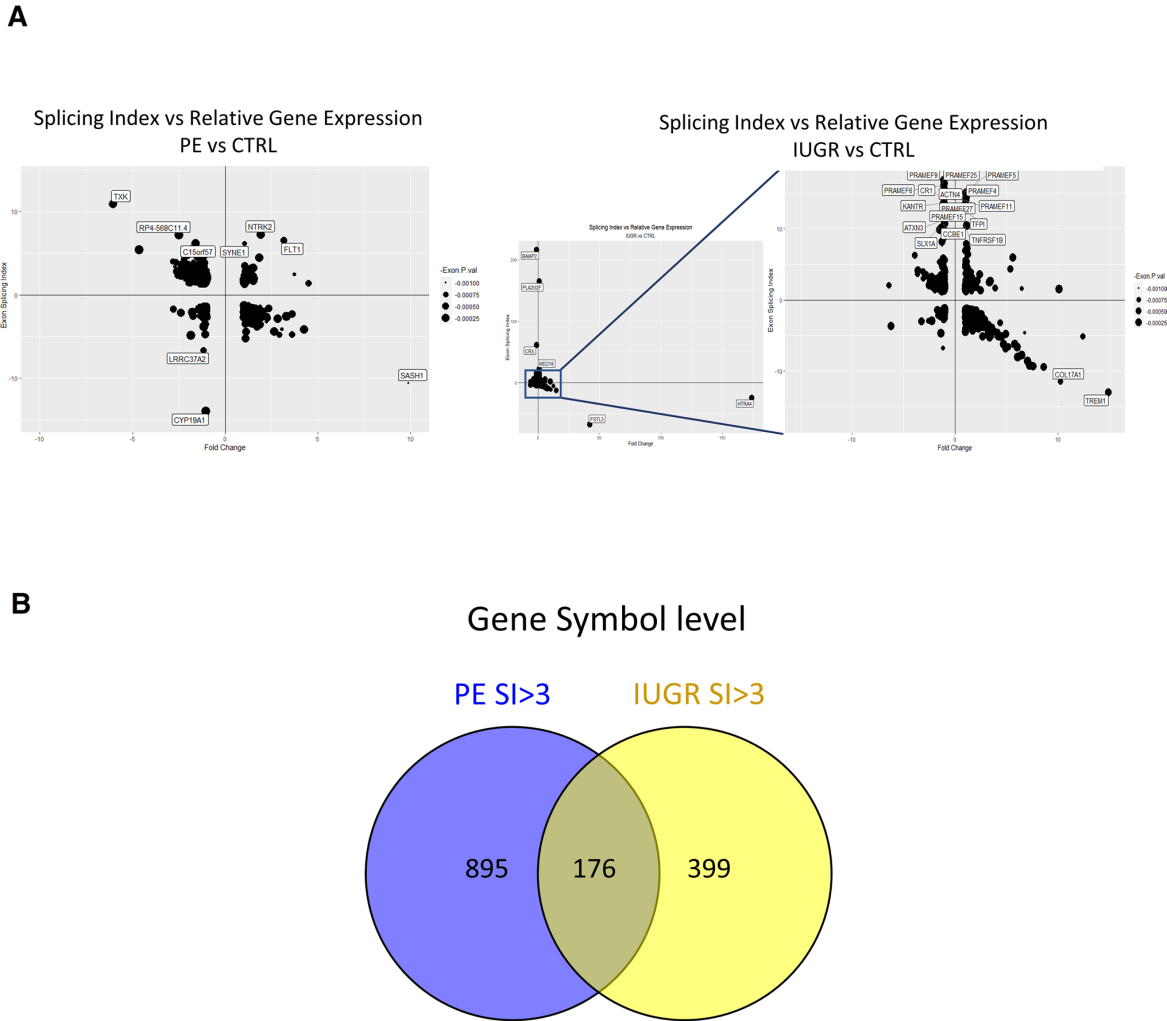
**a** Splicing index represented in function of the fold change of gene expression in PE vs CTRL (left) or IUGR vs CTRL, right. A zoom of the cloud in the blue square is presented. For preeclampsia, gene labels are shown for genes with SI >|6| and for IUGR >|10|. Overall, there was no marked correlation between splicing and gene expression level (see text). **b** A part of the genes only is found both in preeclampsia and IUGR

When using a* p* value threshold of 0.001, 1060 and 1409 genes had a significant splicing index value in PE vs controls and IUGR vs controls, respectively. Splicing index alterations were not correlated with gene expression deregulation between preeclamptic and control samples (Fig. 1a).

For instance, *CYP19A1 or BAIAP2* were not strongly modified at the expression levels in PE placental samples and IUGR samples, respectively, while the splicing index alteration in these genes was amongst the highest (Table 1).

**Table 1.** 48 genes sorted by Splicing Index as modified in pregnancy diseases

Gene	Max SI PE	Max SI PE + IUGR	Max SI IUGR	RT-qPCR validation	Institut Cochin Cohort			Anger Hospital Cohort		Expression 3rd trimester (Pique-Regi et al. 2020)
					Ctrl expression	PE expression	PE + IUGR expression	Ctrl expression	IUGR expression	
BAIAP2	*51.89*	35.61	215.91	No	6.87	6.6	6.59	6.78	6.73	STB
PLA2G2F	*29.19*	22.57	164.47	No	6.1	5.86	6.01	6.14	6.15	
FNDC3B	*19.23*	18.27	1.69	No	19.31	19.31	19.44	19.16	19.15	EVT
RP11-113I22.1	*18.85*	16.99	-1.46	No	4.3	4.22	3.95	4.38	4.26	
CLDN1	*15.51*	9.17	4.25	Yes	15.54	11.55	11.79	15.67	13.4	Endometrial
HMHA1	*11.65*	4.31	7.84	No	3.46	3.61	3.82	3.45	3.58	
FLT1	*11.27*	5.43	-6.24	Yes	17.8	19.47	19.7	17.36	19.89	EVT
TXK	*10.9*	16.7	4.67	Yes	11.17	8.57	8.55	11.54	10.24	
CA10	*10.84*	7.1	3.14	Yes	3.88	3.7	3.41	10.3	8.94	
CPXM2	*10.64*	10.81	4.11	Yes	12.48	9.7	10.54	12.13	10.82	
CDY7P	*9.14*	− 1.64	-1.07	No	4.27	4.32	4.43	4.17	4.03	
FLT4	*9.05*	8.72	6.42	No	10.09	9.27	9.65	9.83	10.16	LED (Lymhoid Endothelial Decidual cells)
ACER3	*8.51*	− 9.21	5.99	No	13.58	14	13.19	13.3	14.01	
STAT4	*8.37*	6.48	2.63	No	7.37	6.34	6.18	7.46	6.79	
PSMD13	*8.35*	3.39	3.31	No	12.96	12.96	12.49	12.81	12.6	
FAM47E; FAM47E-STBD1; STBD1	*8*	7.05	1.86	No	5.56	5.14	5.22	5.56	5.43	
SPX	*8*	− 7.01	7.15	No	8.14	5.92	6.41	8.16	5.52	
NCKAP1	*7.86*	2.85	− 2.5	No	13.81	13.63	13.84	13.57	13.5	EVT
NTRK2	*7.26*	4.1	− 4.44	Yes	5.14	6.06	5.51	4.62	6.3	
SPP1	*7.16*	2.19	− 3.48	No	15.54	13.15	15.73	15.86	15.22	Macrophage
TNFRSF1B; MIR4632; MIR7846	*7.03*	5.39	10.42	Yes	8.37	8.83	9.03	8.96	9.14	
EIF1AY	*6.93*	− 11.93	5.64	No	8.16	6.46	10.75	8.05	7.35	VCT
CYP19A1	**− 68.93**	− 5.02	3.56	No	14.08	13.08	13.42	19.92	19.92	CTB
PSG4	**− 31.84**	− 28.56	3.8	No	19.91	19.85	19.88	19.89	19.93	STB
XIST	**− 24.17**	298.39	− 24.55	No	13.95	16.16	6.71	12.71	17.02	
FSTL3	**− 21.76**	− 16.73	− 67.97	No	8.03	11.87	11.72	7.06	12.45	EVT
RPSA; SNORA62; SNORA6	**− 20.35**	2.81	− 1.67	No	15.46	15.39	15.39	15.19	15.33	npiCTB (non-proliferative interstitial cytotrophoblast)
GABRE; MIR224; MIR452	**− 20.35**	− 2.44	− 8.66	No	16.08	15.37	16	16.49	15.99	CTB
SH3BP5	**− 14.23**	− 2.01	− 4.91	No	8.95	8.88	8.97	8.09	10.15	EVT
PDXDC2P	**− 13.05**	− 5.63	2.27	No	12.29	11.82	12.28	13.08	12.76	
BIN2	**− 10.91**	− 11.55	− 8.01	No	6.42	7.17	6.81	8.84	11.45	
SASH1	**− 10.59**	− 16.6	− 9.28	No	7.96	11.27	11.2	8.01	10.93	LED
LGALS8	**− 9.52**	− 5.48	2.31	No	14.39	13.58	14.09	14.77	14.47	
ST18	**− 9.36**	3.05	− 3.69	No	3.37	3.47	3.26	3.25	3.19	
HTRA4	**− 9.29**	− 12.66	− 24.88	No	10.91	15.72	16.87	10.23	17.67	EVT
ATG2B	**− 9.11**	− 2.92	1.87	No	8.33	8.06	8.57	8.34	8.39	
DDX17	**− 8.9**	− 1.77	2.02	No	16.78	16.58	16.73	16.82	16.84	EVT
CAP2	**− 8.58**	− 5.82	1.92	No	14.36	13.62	13.76	14.59	14.14	STB
BCAP29	**− 6.6**	− 2.59	2.67	No	13.53	13.4	13.26	12.98	13.38	LED
ORC4	**− 6.17**	− 3.95	1.62	No	11.53	10.81	11.52	11.13	11.14	
SLC6A10P	**− 6.11**	− 4.13	− 8.59	No	8.96	11.13	10.51	8.56	11.39	
P4HA1	**− 6.08**	− 6.88	− 4.86	No	8.83	10.65	11.08	8.79	10.47	EVT
LYRM5	**− 6.06**	− 7.47	− 1.84	No	8.5	8.26	9.06	6.13	5.02	
DPP4	**− 5.94**	1	− 1.01	No	5.95	6.37	6.4	6.42	6.42	CTB
ACOXL	**4.46**	6.24	3.64	Yes	9.12	7.47	7.06	9.35	8.1	
TIE1	**4.01**	5.13	− 1.66	Yes	6.19	6.49	6.4	6.08	6.29	LED
LGALS14	**− 2.22**	− 2.42	− 2.02	Yes	19.9	19.7	19.55	19.92	19.58	STB
LEP	**− 1.91**	− 2.54	− 1.56	Yes	11.85	12.79	13.1	7.42	15.28	STB

#### Alternative splicing concerns specific gene categories in PE and IUGR

**Fig. 2:**
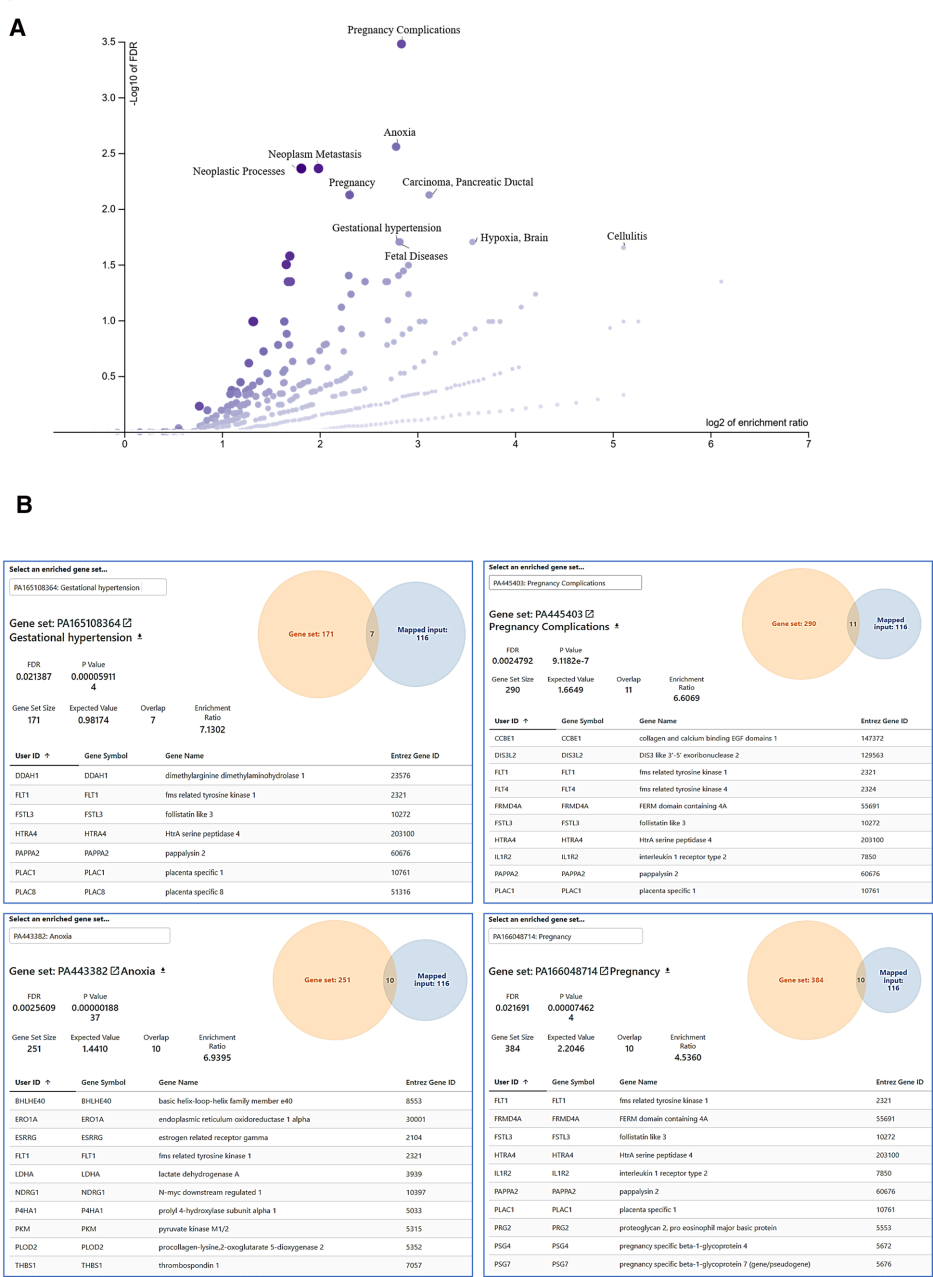
WebGestalt analysis of the genes that are alternatively spliced in common in the two diseases under scrutiny, in comparison with the disease database GLAD_4U. The database recognized 116 genes among the 176 submitted. **a** The enrichment in genes in the group is represented on the X-axis, while the FDR is represented on the Y-axis. The most significant pathways (with FDR < 0.05) are identified. **b** Highly relevant gene sets are represented. As an example of how to read the figure, the first on the upper left means that 178 genes are in the geneset ‘Gestational Hypertension’, 7 of which are in common with the 116 that constitute the input (the 116 that were identified out of the initial input of 176). This enrichment is highly significant (7.1 more genes that expected, FDR = 0.0214)

Since *p* value allows to pinpoint significant but also small variations in splicing (that may not be relevant), we decided to focus on transcripts with SI >|$3$|, a list marginally different from the one given with the p < 0.001 threshold. We identified a list of 1456 transcripts in PE vs control placentas, and 725 transcripts in IUGR vs control placentas at the same threshold. Among those, 1071 and 575 have an official gene symbol (Supplementary Tables S3 and S4) and 176 were found alternatively spliced in both diseases (Fig. 1b). These latter 176 genes encoded proteins that constituted a network of Protein–Protein Interactions (*p* = 0.00097, Supplemental figure S1, String database, https://string-db.org/cgi (Szklarczyk et al. 2019)), showing that the splicing alteration does not occur randomly in the placental genome in pathological situations. We then performed an over-representation analysis using WebGestalt (Wang et al. 2017; Liao et al. 2019) on the 176 genes found alternatively spliced in both disease conditions. We compared our gene list with the GLAD4U database (Jourquin et al. 2012), that collects gene sets associated with diseases. We found a significant enrichment for Pregnancy complications, fetal diseases, gestational hypertension, hypoxia and several cancer pathways (Fig. 2a). The exhaustive values and genes involved are presented as Fig. 2b for ‘Gestational Hypertension’, ‘Pregnancy complications’, ‘Anoxia’, and ‘Pregnancy’.

#### Functional clustering of alternatively spliced genes partly differs between PE and IUGR

Then we proceed to separately analyze the genes spliced in either IUGR or PE. We found that while the two pathologies shared biological processes related to secretion, exocytosis and vesicle metabolism, substantial clustering differences were observed.

In IUGR (Table 2), KEGG and Reactome pathways related to hypoxia, steroidogenesis, hormone metabolism and anion transport were enriched. Against disease database (GLAD4U and OMIM), the alternatively spliced genes in IUGR were widely associated to 'pregnancy complications', 'Nitric Oxide metabolism' (known as intimately linked to placental pathology) (Aouache and Biquard 2018; Motta-Mejia et al. 2017; Dymara-Konopka and Laskowska 2019), 'preeclampsia' and 'eclampsia', but also to autoimmune diseases, especially 'lupus erythematosus' (SLE), which is consistent with the increased risk of IUGR and other defective pregnancy outcomes documented in autoimmune patients, especially those affected by SLE (Do and Druzin 2019).

**Table 2:** Enrichment analysis of genes differentially spliced in IUGR

Gene set	Description	Expect	Ratio	*p* value	FDR
Gene ontology biological processes					
GO: 0,046,903	Secretion	33.803	1.8638	8.58E–07	0.006826
GO: 0,009,628	Response to abiotic stimulus	23.651	2.0295	2.16E–06	0.006826
GO: 0,032,940	Secretion by cell	31.002	1.8709	2.25E–06	0.006826
GO: 0,045,055	Regulated exocytosis	16.491	2.1831	9.5E–06	0.017593
GO: 0,071,363	Cellular response to growth factor stimulus	13.9	2.3021	1.07E–05	0.017593
GO: 0,009,719	Response to endogenous stimulus	33.592	1.7564	1.28E–05	0.017593
GO: 0,009,725	Response to hormone	20.261	2.0236	1.37E–05	0.017593
GO: 0,002,576	Platelet degranulation	2.6747	4.4864	1.55E–05	0.017593
GO: 0,006,820	Anion transport	12.321	2.3538	1.86E–05	0.018812
GO: 0,070,848	Response to growth factor	14.532	2.202	2.57E–05	0.023392
KEGG pathways					
hsa04913	Ovarian steroidogenesis	1.1546	6.9286	1.6E–05	0.003405
hsa04066	HIF-1 signaling pathway	2.3564	4.6681	2.09E–05	0.003405
hsa04923	Regulation of lipolysis in adipocytes	1.2725	5.5012	0.000249	0.027062
hsa04014	Ras signaling pathway	5.4669	2.7438	0.000359	0.029234
Reactome pathways					
R-HSA-114608	Platelet degranulation	3.1046	3.8652	6.28E–05	0.048135
R-HSA-76005	Response to elevated platelet cytosolic Ca2+	3.2249	3.721	9.1E–05	0.048135
R-HSA-202733	Cell surface interactions at the vascular wall	3.2971	3.6395	0.000113	0.048135
R-HSA-879518	Transport of organic anions	0.2888	13.85	0.000139	0.048135
R-HSA-209822	Glycoprotein hormones	0.2888	13.85	0.000139	0.048135
Disease_GLAD4U					
PA445403	Pregnancy complications	5.0666	5.5264	2.53E–13	3.80E–10
PA166048714	Pregnancy	6.7088	4.7698	2.79E–13	3.80E–10
PA165108364	Gestational hypertension	2.9875	6.3598	1.73E–10	1.57E–07
PA445398	Pre-Eclampsia	2.8128	5.6882	2.44E–08	1.45E–05
PA443984	Eclampsia	2.8303	5.6531	2.67E–08	1.45E–05
PA445058	Neoplasm metastasis	11.409	2.8049	1.67E–07	7.58E–05
PA166048749	Pregnancy third trimester	1.1531	8.6724	2.07E–07	8.02E–05
PA165108943	Retained placenta NOS	0.47171	14.839	3.08E–07	0.000105
PA446021	Vascular diseases	8.5782	3.0309	5.96E–07	0.00018
PA166048848	Menopause	2.7429	5.104	7.00E–07	0.00019
Disease_OMIM					
611,162	Malaria, susceptibility to malaria, resistance to, included	0.076176	52.51	6.48E–07	4.28E–05
152,700	Systemic lupus erythematosus	0.035848	55.792	0.000523	0.017249
609,423	Human immunodeficiency virus type 1, susceptibility to	0.067214	29.756	0.001924	0.028948
601,665	Obesity, leanness included	0.067214	29.756	0.001924	0.028948
415,000	Spermatogenic failure, y-linked, 2	0.071695	27.896	0.002193	0.028948

The same type of analysis applied to the PE-alternatively spliced genes yielded quite different results (Table 3). In terms of Biological processes, extracellular matrix, circulatory system and several neuron/axon ontology terms were found enriched specifically in PE. KEGG and Reactome pathways consistently pointed out to extracellular matrix organization pathways. In terms of diseases databases (GLAD4U and OMIM), ‘pregnancy’ and ‘pregnancy complications’ appeared enriched, but also, interestingly, numerous pathological pathways implicated in neurodevelopmental diseases emerged with GLAD4U ('Brain diseases', 'CNS diseases', 'Dementia', 'Cri-du-Chat syndrome'), while OMIM pointed out to SLE (Qing et al. 2011) and Malaria susceptibility, consistently with the IUGR OMIM terms. In this case, nevertheless, an enrichment in genes involved in Alzheimer's disease was also found, consistently with the GLAD4U keywords.

**Table 3:** Enrichment analysis of genes differentially spliced in PE

Gene Set	Description	Expect	Ratio	*p* value	FDR
Gene ontology biological processes					
GO: 0,016,192	Vesicle-mediated transport	70.148	1.6394	4.34E–08	0.000199
GO: 0,030,198	Extracellular matrix organization	12.534	2.7924	4.37E–08	0.000199
GO: 0,043,062	Extracellular structure organization	14.449	2.4916	4.80E–07	0.001455
GO: 0,006,887	Exocytosis	32.293	1.889	1.25E–06	0.002845
GO: 0,072,359	Circulatory system development	37.061	1.8078	1.7E–06	0.003095
GO: 0,031,102	Neuron projection regeneration	1.8783	5.3239	1.41E–05	0.021369
GO: 0,031,103	Axon regeneration	1.5532	5.7944	1.85E–05	0.024045
GO: 0,048,678	Response to axon injury	2.4201	4.5452	2.55E–05	0.028951
GO:0,035,295	Tube development	35.832	1.7024	3.19E–05	0.030548
GO: 0,045,055	Regulated exocytosis	28.283	1.8032	3.38E–05	0.030548
KEGG pathways					
hsa04510	Focal adhesion	7.8332	2.6809	3.33E–05	0.010845
hsa04512	ECM-receptor interaction	3.2277	3.408	0.000337	0.05495
hsa04960	Aldosterone-regulated sodium reabsorption	1.4564	4.8063	0.000507	0.055129
Reactome pathways					
R-HSA-1474244	Extracellular matrix organization	11.893	2.8589	2.91E–08	5.03E–05
R-HSA-216083	Integrin cell surface interactions	3.3584	4.7641	1.70^E^–07	0.000147
R-HSA-114608	Platelet degranulation	5.0969	2.9429	0.000168	0.078328
Disease_GLAD4U					
PA443275	Adhesion	22.33	2.4183	2.04E–09	5.55E–06
PA443553	Brain diseases	16.179	2.2251	7.22E–06	0.008322
PA166048714	Pregnancy	11.194	2.5013	9.18E–06	0.008322
PA443657	Central nervous system diseases	16.674	2.159	1.39E–05	0.009476
PA444447	Carcinoma, hepatocellular	11.077	2.4374	2.1E–05	0.011403
PA443853	Dementia	8.1915	2.6857	2.83E–05	0.012844
PA445403	Pregnancy complications	8.4538	2.6024	4.57E–05	0.016622
PA166123006	interstitial fibrosis	2.128	4.6992	4.89E–05	0.016622
PA443812	Cri-du-chat syndrome	0.46642	10.72	6.92E–05	0.020562
PA445080	Neovascularization, pathologic	6.3841	2.8195	8E–05	0.020562
Disease_OMIM					
146,110	Hypogonadotropic hypogonadism 7 with or without anosmia	0.11153	35.866	3.14E–06	0.000207
611,162	Malaria, susceptibility to malaria, resistance to, included	0.13542	29.537	7.35E–06	0.000242
152,700	Systemic lupus erythematosus	0.063729	31.383	0.001671	0.036762
104,300	Alzheimer disease	0.087628	22.824	0.003234	0.053355

In summary, this comprehensive description of abnormal splicing in IUGR and PE reveals quite different enrichment of alternatively spliced genes between two important placental diseases and normal placentas. Notably, a connection with neurological disease genes was exclusive to PE. Comparing our sQTLs results, with placental eQTLs identified in previous studies (Peng et al. 2017), we found four genes in common amongst the cis-eQTLs (ACER3, CLDN1, PSG4, LGALS8). A contingency chi2 revealed a marginally significant enrichment (12.8% expected versus 33% observed, *p* = 0.034).

### Individual validation of alternative splicing on an enlarged collection of placental samples

**Fig. 3:**
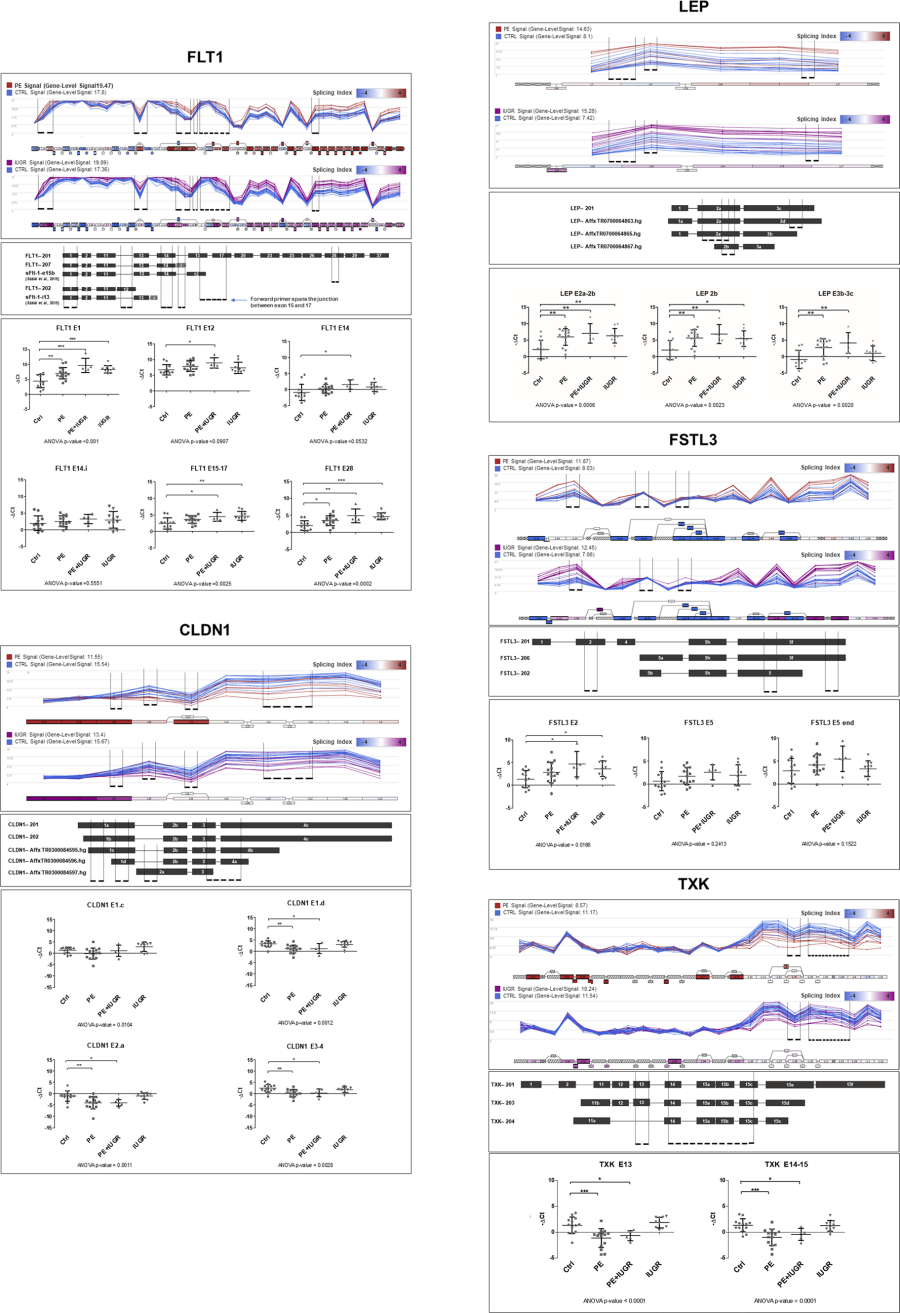
Locus-specific RT-qPCR analysis of genes that were found modified in pathological placentas. Each panel represents the splicing profile of a gene. For instance, for FSTL3 there is an increased splicing index in the 5′ and 3′ regions of the gene, in preeclampsia versus controls and IUGR vs controls (two upper graphs), drawn with the positions of the primer couples that were used for the RT-qPCR. Significant alterations are in this case essentially detected in the E2 tested region, while the others are not significant, consistently with the microarray graph

We next validated some of these alterations through a targeted approach, based upon exon-specific primers and RT-qPCR. For this validation, we selected 12 genes based upon their known involvement in preeclampsia and/or their very high splicing index differences between healthy and preeclamptic placentas: FLT1, CLDN1, LEP, FSTL3, TXK, CAP2, CA10, TNFRSF1B, ACOXL, TIE1, LAGLS14, CPXM2. Maximal splicing indexes (p values) were respectively: FLT1 11.27 (*p* = 0.0016), CLDN1 15.51 (*p* = 0.0063), LEP 2 (0.2957), FSTL3 − 21.76 (*p* = 0.005), TXK 10.9 (*p* = 8.55E^−05^), CAP2 − 8.58 (*p* = 0.0331), CA10 10.84 (*p* = 0.0557), TNFRSF1B 7.03 (*p* = 0.0035), ACOXL 4.46 (0.0241), TIE1 4.01 (*p* = 0.0034), LGALS14 − 2.22 (*p* = 0.5275), CPXM2 10.64 (*p* = 0.0301). Additional new samples were included along with the samples used in the microarray analysis, for a final total of 13 controls, 15 isolated PE, 5 PE + IUGR and 10 isolated IUGR samples. Overall, we confirmed splicing alterations in diseased placentas for 10 out of 12 genes (except LGALS14 and CPXM2). An example of analysis is given for five genes (FLT1, CLDN1, LEP, FSTL3 and TXK) in Fig. 3, and the remaining genes are presented as Supplementary figures S2.

### Genetic regulation of alternative splicing in the human placenta—determination of sQTLs

We next analyzed the genetic basis of splicing alterations in the different individual placentas, focusing upon a sample of 48 genes with the most differential splicing index between normal and preeclamptic placentas (Table 1). Several of these genes are well known from the literature to be modified at the expression level between PE and normal placentas such as FLT1, LEP, PAPPA2, HTRA4 (Vaiman et al. 2013). Nineteen out of the 48 genes were also significantly modified at the expression level between controls and IUGR, as assessed by the analysis of the Angers Hospital-IUGR cohort. By PCA analysis, we could show that there was no major effect of the batch (Supplementary Figure S3).

#### Splicing is influenced by variants located in the vicinity of genes (cis-sQTL) and at far locations (trans-sQTL)

**Fig. 4:**
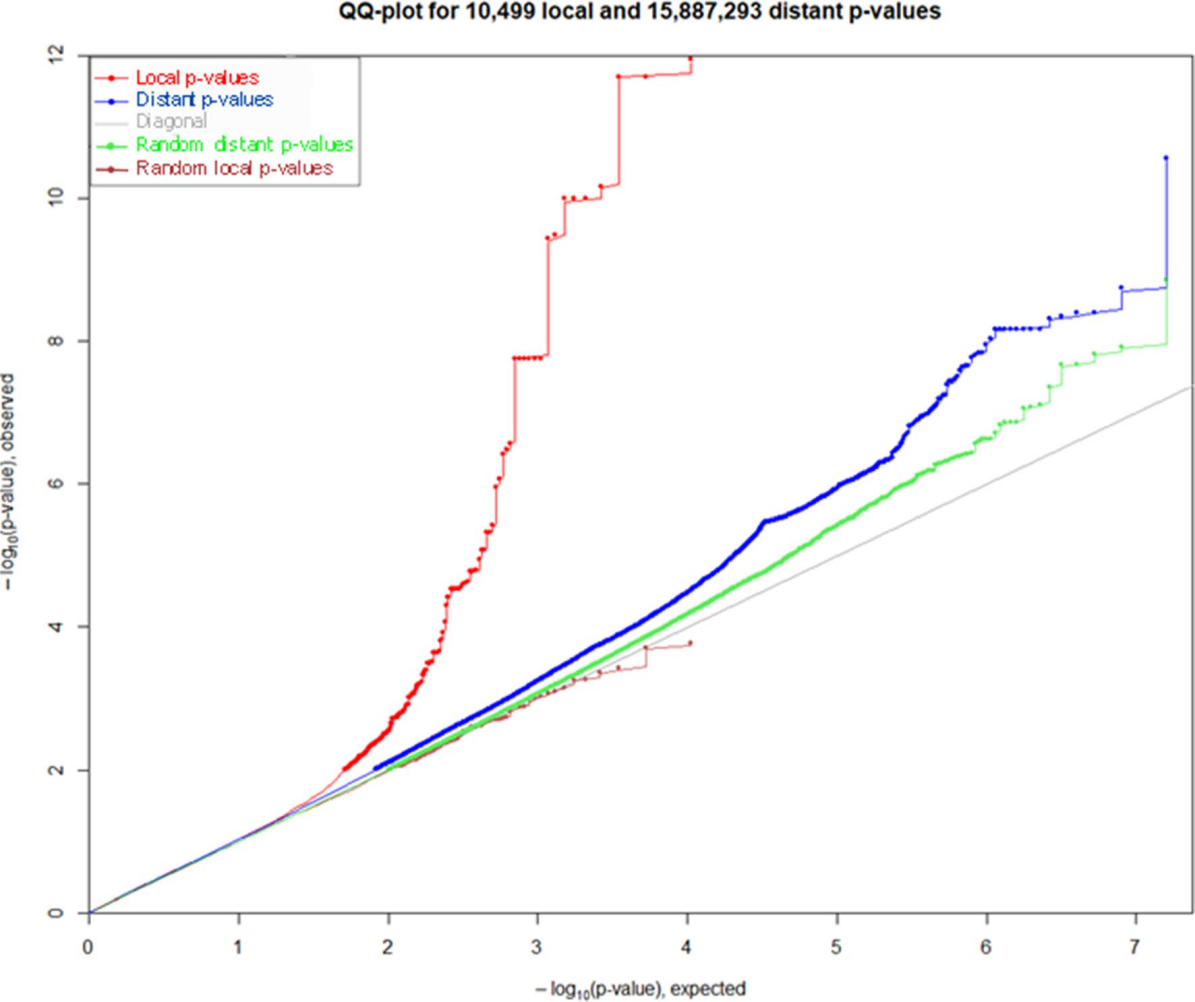
QQ-plot measuring the discrepancy between real data and expected data from random (grey line)

Each placental DNA was genotyped using a SNP genotyping array that encompasses ~ 710,000 SNPs and MatrixeQTL R package was used to investigate the impact of SNP variants on individual splicing indexes (Shabalin 2012). Placental Individual Splicing Indexes (ISIs) were computed along with the genotype data. A QQ-plot (Fig. 4) was obtained and allowed the identification of 180 cis-sQTLs (1 Mb around the gene under scrutiny) with *p* < 0.01 and 52 with FDR < 0.05 and 199,884 trans-sQTLs (> 1 Mb) with *p* < 0.01 and 52 with FDR < 0.05. The detailed list of cis-sQTLs identified is given as Supplementary Table S5. To add stringency to the approach, we re-ran the program focusing only on the control samples (Tong et al. 2018). Despite the loss of power due to this reduced size, we werestill able to detect 5 cis-sqtl with a FDR < 0.0.5 that are all common to the complete list and presented in bold in Table S5.

#### Cis-sQTLs have additive effects on splicing

**Fig. 5:**
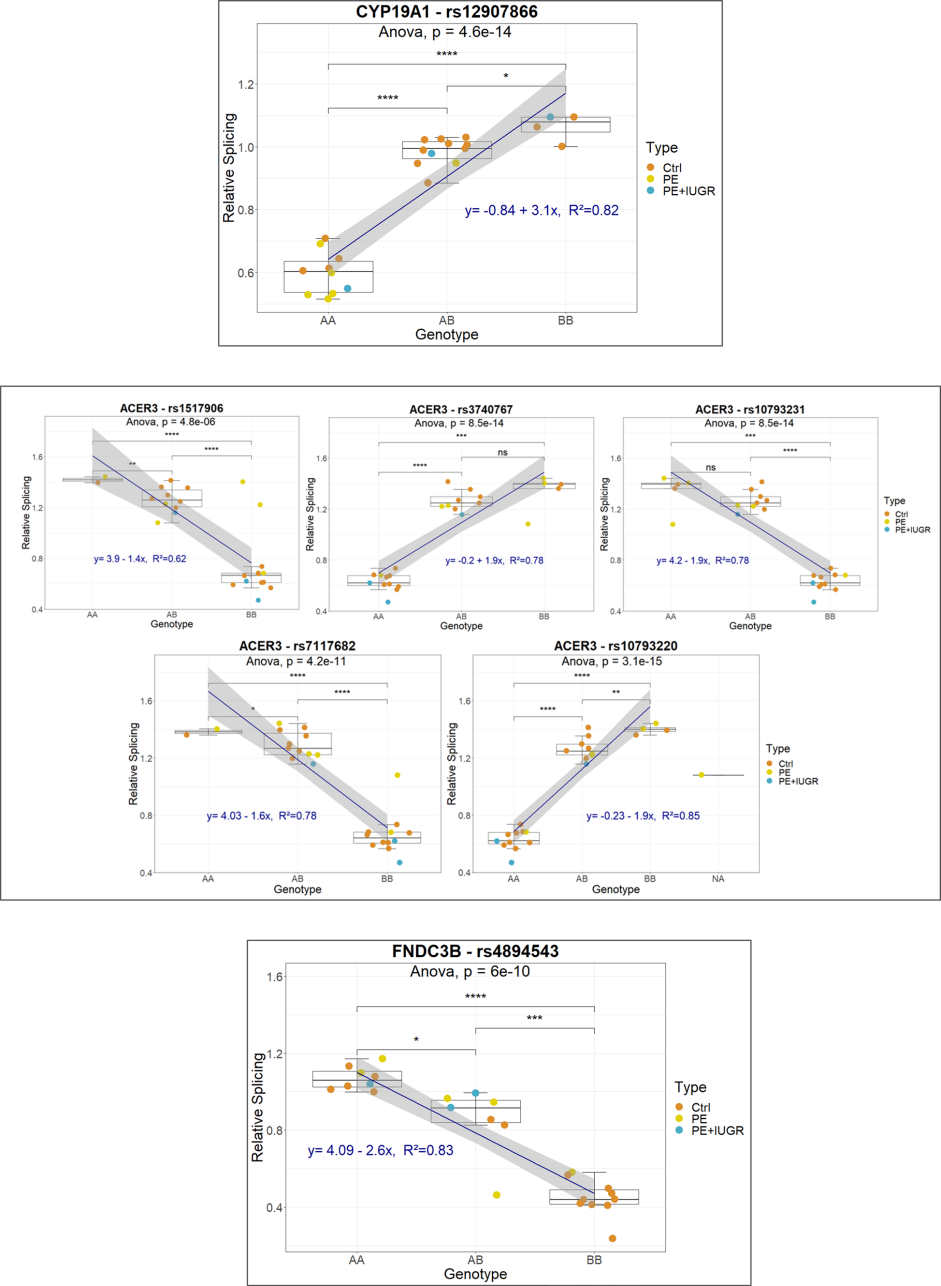
Analysis of genotype versus splicing at the individual level for cis sQTLs in the placenta

A selection of highly significant cis-sQTLs are presented in Fig. 5. It is interesting to note that in these cases, the splicing effect is overall linear in function of the occurrence of one of the alleles (additive effect). For instance, in the first example (CYP19A1-rs12907866), the splicing index is the lowest in AA genotypes, intermediate in AB and higher in BB. The same type of profile is visible in the other examples presented. These influences on SI do not appear to be strictly connected to the disease status. Cis-sQTLs may induce alternative splicing by influencing the binding of splicing factors, by modifying the secondary structure of mRNAs, or any other local influence. However, they are not giving an image of the alternative splicing regulation at the genome level. Thus, to identify variants that influence splicing distantly from their location, we studied in more detail trans-sQTLs.

#### Trans s-QTL analysis reveals major loci associated to splicing in the placenta

The conventional significance threshold for a genome-wide analysis (*p* < 10^–8^) identified 52 significant SNP-gene couples for trans-sQTL (Supplementary Table S6). At *p* < 0.001, 24,867 QTLs were found, while 3,639 QTLs passed the *p* < 0.0001 threshold. As reported in many studies, keeping an FDR < 0.05 or a *p* < 10^–8^ will miss QTL with biological relevance (Morrow et al. 2018). Therefore, we developed a novel approach not to lose this relevant biological information; in particular, we were interested in identifying bandmaster loci that would control the splicing of a large part of the 48 genes that we identified as the most strongly spliced.

To perform this, we organized the dataset by sorting the SNPs along the chromosomes to find isolated SNPs (or windows of SNPs separated by less than 2000 bp—a distance in the range of minimal chromosomal block sizes of markers in linkage disequilibrium (Pritchard and Przeworski 2001)) that were significantly associated to alternative splicing of more than one gene.

**Fig. 6:**
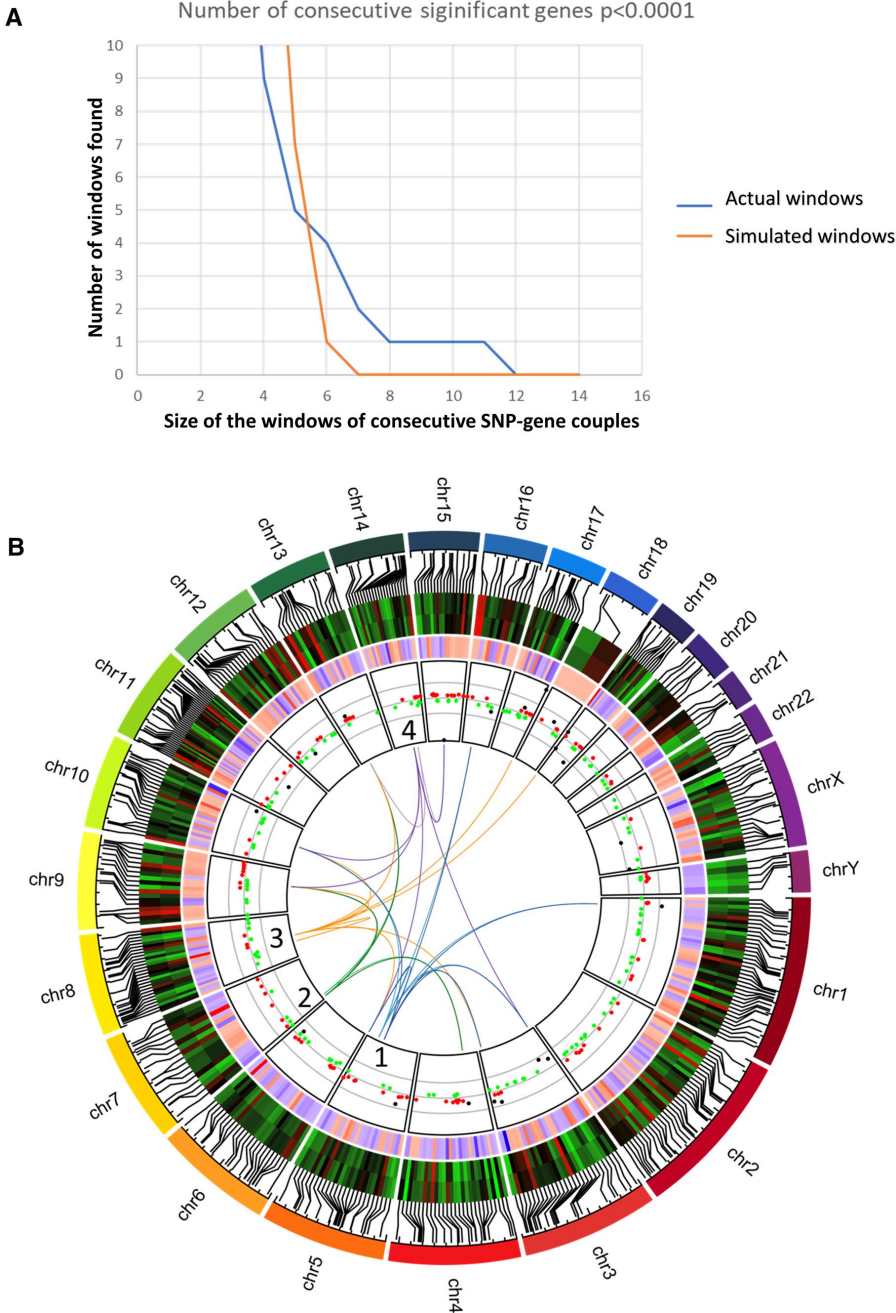
Presentation of trans-sQTL. **a** Monte-Carlo analysis of windows of consecutive trans sQTL in the placentas. Trans sQTL were defined at a threshold of *p* < 0.0001 (see text). In the windows computed from the actual dataset (blue line) the longest found was of 11 consecutive SNP-genes. In the simulated windows, the longest found was composed of 6 consecutive SNP-genes (4 such in the actual dataset). Overall, while one > 5 window was found in the simulated data, 9 were found in the actual dataset. **b** circus-plot presenting the four trans-sQTl regions analyzed further in the study. Tracks are numbered from the outer to the inner part of the plot. 1st track: representation of each chromosome. 2nd track: location of the 467 genes used in the 3rd track. 3rd track: heatmap of the splicing indexes in PE and Ctrl samples for the 467 genes presenting a standard deviation equal or higher than 0.5: (Red = 19.42, Green = 3.82). 4th track: ratio of downregulation/upregulation of the 467 genes between PE and Ctrl (Red: > 3 (increased in PE), Blue < − 3 (increased in controls). 5th track: Splicing index of the 286 most differentially spliced genes observed in TAC comparing Ctrl vs PE (red dots = gene with SI between 10 and 3.99, green dots = genes with SI between − 3.99 and − 10, black dots = genes with SI higher than 10 or lower than − 10). 6th track: constant trans-eQTLs. Blue (1) SNPs located in ARHGAP26/AS; Green (2) SNPs located in LOC105375161; Orange (3) SNPs located in LOC105375897; Purple (4) SNPs located in GPHN/LOC105370538. The other side of the line marks the location of the gene in which the relative splicing index is affected by the corresponding SNP

A Monte-Carlo statistical analysis was performed to evaluate the longest possible windows of consecutive SNP-gene couples obtained from a random organization of the SNPs (simulated windows). At a threshold of 0.001, the maximal window length was of 19 consecutive gene-SNP couples, while random cases lead to a maximal size of 10 gene-SNP couples. Performing the same operation at the 0.0001 threshold we identified a maximum window size of 6 in the randomized dataset (one occurrence), while nine windows of more than 6 consecutive SNP-gene couples (Fig. 6a) were identified from the real dataset. The nine windows identified are located on chromosomes 2, 5, 7, 8, 10, 13, 14 (at two locations) and 21. To note, four of these windows were also detected at the threshold of 0.001: on chromosomes 5, 7, 8, and the second region on chromosome 14. Also, two of these windows (on chromosome 2 and 14) encompassed gene-SNPs couples that were significant at a genome-wide threshold (*p* < 10^–8^, rs13006826-SLC6A10P and rs9323491-CYP19A1). We decided to focus on these four regions, presented in Fig. 6b as the blue, green, orange and purple fountains of a circus Plot (Gu et al. 2014).

The Chromosome 5 region contains two SNPs separated by 702 bp, rs13185255 and rs12520828. These SNPs are located inside the ARHGAP26 gene, or its antisense and are associated with increased splicing alterations for FLT4, CLDN1, PLA2G2F, SH3BP5, P4HA1 and SLC6A10P.

The Chromosome 7 region spans 314 base pairs and encompasses 2 SNPs (rs6964915 and rs6965391). These SNPs are located inside the LOC105375161 non-coding RNA, which harbors the highest level of expression in the placenta ((https://www.ncbi.nlm.nih.gov/gene/105375161) and (Fagerberg et al. 2014)). RT-qPCR results interrogating two distinct regions of LOC105375161 showed no difference in expression between the sample groups (Supplementary Fig. S5). The splicing alterations of 3 genes were found associated with variants in this Chr7 region (FLT1, TXK and NTRK2).

The Chromosome 8 region encompasses a unique SNP (rs1431647) located inside the non-coding RNA LOC105375897, which is expressed at a low level in the placenta. Eight genes were potentially affected by this variant at the splicing level (FSTL3, FLT1, P4HA1, NTRK2, FLT4, BAIAP2, CLDN1, ST18). By RT-qPCR, we could show that LOC105375897 is expressed at similar levels between control and preeclamptic placentas, while is overexpressed significantly in IUGR ~ 30 fold (*p* value < 0.01) (Supplementary Fig. S5).

The Chromosome 14 region spans 828 bp and encompasses two SNPs, rs7145295 and rs7151086 that are located inside the GPHN gene (harboring the non-coding RNA LOC105370538, which was not detectable in our samples by RT-qPCR). Six genes were potentially affected at the splicing level: CYP19A1, FLT1, P4HA1, FLT4, SH3BP5, and NTRK2).

**Fig. 7:**
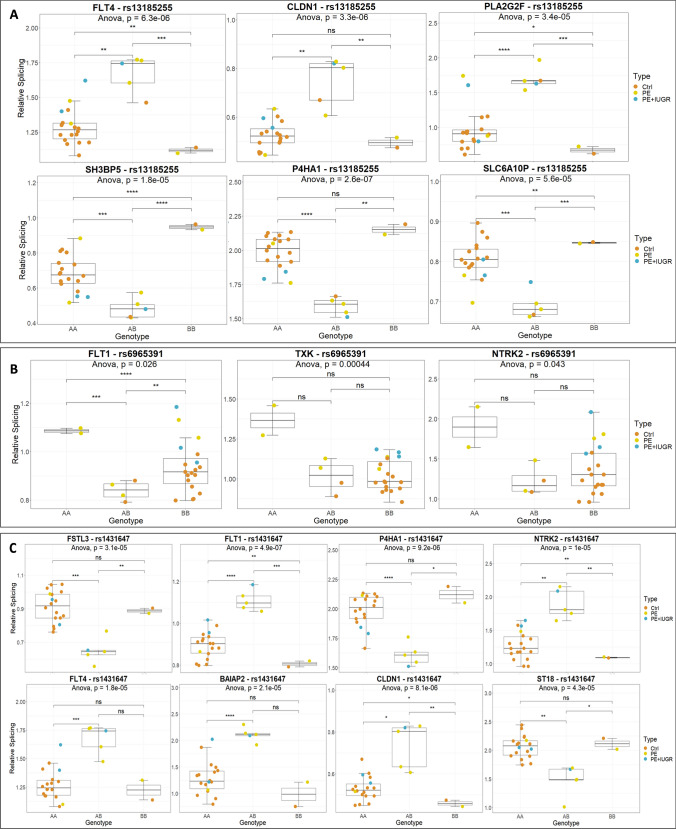
Presentation of trans-sQTLs data for three SNPs found in the regions presented in Fig. 6. **a** rs13185255 from the chromosome 5 trans-sQTLs, **b** rs6965391 from the chromosome 7 trans-sQTLs, **c** rs1431647 from chromosome 8. **d** rs7145295 and rs7151086 from chromosome 14

The association between these SNPs and splicing levels is represented in Fig. 7. The two SNPs identified for chromosome 5 as well as for chromosome 7 had the same profile, and thus only one SNP of each region was represented. For rs13185255 it was the heterozygous genotype that was different from the homozygous genotypes (Fig. 7a). There were no obvious association between the levels of splicing, the genotypes of the QTL and the status of the patient. For rs6965391 the AA genotype was characterized by a higher splicing index for the three target genes (Fig. 7b), with a possible association with preeclampsia but that should be confirmed on a larger sample since the AA genotype was the rarest. For rs1431647, it was also the heterozygous genotype that was different from the others, without obvious connections with the disease status (Fig. 7c). In Fig. 7d are represented the two SNPs characterizing the fourth region studied. One of the SNPs (rs7145295) presents an additive behavior in terms of splicing levels for CYP19A1 (*r* = 0.85, *p* < 10^–4^). AA was marginally associated with preeclampsia (Chi-Square, *p* = 0.022, Log-Likelihood, *p* = 0.029), and the data are consistent with the second SNP nearby rs7151086. In both cases, a higher splicing index characterizes the BB genotype.

## Methods

### Ethical statements and sample collection

The placenta collections have already been described elsewhere (Gascoin-Lachambre et al. 2010; Ducat et al. 2019).

Cochin Hospital cohort: placentas were collected from two maternity wards (Hôpital Cochin and Institut de Puériculture, Paris, France). This study was approved by the Ethics Committee and CCPPRB (Comité Consultatif de Protection des Personnes dans la Recherche Biomédicale) of Paris Cochin. Placentas were obtained from cesarean sections before the onset of labor. Exclusion criteria were diabetes, chromosomal and fetal malformations, maternal infections, and addictions. The inclusion criteria used for pre-eclampsia were systolic pressure above 140 mmHg, diastolic pressure above 90 mmHg and proteinuria above 0.3 g per day. Women who underwent a cesarean section without disease during pregnancy formed the control group. All patients gave their written consent for the use of their placenta and blood samples.

Angers University Hospital cohort: collection and use for research purpose (including genetic analyses) of placentas from pregnancies complicated with IUGR or healthy pregnancy have been approved by the Ethics Committee of Angers. The inclusion criteria used for IUGR was weight at birth < 10th percentile. Vascular IUGR was defined by a reduction of fetal growth during gestation, with a notch observed by Echo-Doppler in at least one uterine artery and with Doppler abnormalities on ombilical Doppler and/or cerebral Doppler and/or ductus venosus, and with a birth weight below the 10th percentile according to Audipog growth curves. As for the Cochin cohort, women who exhibited any of the following criteria were excluded from the study: diabetes, pre-eclampsia, chromosomal and fetal malformations, maternal infections, aspirin treatment, and addictions (such as tobacco or drug usage). This collection is registered to the French Ministry of Research under number DC-2011-1467. The processing of personal data implemented as part of the project has been authorized by the French Data Protection Authority (CNIL, no. pWP03752UL). All participants provided written informed consent prior to inclusion. The study was conducted in accordance with the declaration of Helsinki (Chabrun et al. 2019).

The placental sampling was systematically done in placental regions that appeared visually normal.

### RNA and DNA extraction from human placentas

RNA isolation followed classical protocols. The placentas were processed in the 30 min post cesarean section. The fetal membranes were discarded, and then, from two cotyledons to three cotyledons, villous trees were washed thoroughly in PBS and placed in TriZol™. Then the tissue was ground with a metal bead in a tissue grinder in 1.5 ml tubes. Then, 200 µl of chloroform were added, the tubes were centrifuged at 10,000*g* RT, and the supernatant collected, reprecipitated in isopropanol by centrifugation. The pellet was then washed in 70% ethanol, dried under a fume hood, and resuspended in RNAse-free water. The quality of the RNA was evaluated by 2100 bioanalyzer (Agilent). Samples with RIN > 7 were used for microarray analysis and RT-qPCR. DNA was prepared from the same sample by standard lysing protocols (Proteinase K treatment overnight at 55 °C), spooling of the DNA, washing with ethanol 70%, resuspension I TE (1–0.1) at ~ 200 ng/µl.

### Transcriptomics and genotype datasets

One hundred ng of RNA from each placental sample were analyzed by ClariomD (Affymetrix) microarray assay. This tool interrogates 134,748 probes, including coding genes (18,858), non-coding genes (66,845) and genes deduced by bioinformatics analysis (10,001), in addition to pseudogenes, small RNA and ribosomal RNAs. Overall, more than 540,000 transcripts are analyzed with this tool. With the TAC tool, a splicing index can be calculated and a statistical value associated to it. The splicing index was calculated by the Transcriptome Analysis Console (TAC) from Affymetrix, using the following formula:

1 $${\text{Log}}2\left[ {\frac{{\left( {\frac{{{\text{probeset1}}\;{\text{intensity}}}}{{{\text{Gene1}}\;{\text{intensity}}}}} \right)}}{{\left( {\frac{{{\text{probeset1}}\;{\text{control}}\;{\text{intensity}}}}{{{\text{Gene1}}\;{\text{control}}\;{\text{intensity}}}}} \right)}}} \right].$$

The probeset is defined by the fluorescence of a given probe (either a junction probe, or an exon-specific probe), present in the array. More details can be obtained in (Jimeno-Gonzalez et al. 2015).

To be able to evaluate the splicing variation on an individual basis, we first defined an individual splicing index for each sample (Individual splicing index—ISI). Inside a given gene and for a given placental sample, we obtained the different fluorescent measures for each of the probes spanning the gene. We then selected the probe with the highest splicing index and divided its fluorescent signal level by the geometric mean of the complete group of probes. For a given placental sample, the relative splicing index for Gene A is therefore given by

2 $$\frac{{{\text{MAX}}\left( {{\text{FprobeGAi}}} \right)}}{{\sqrt[n]{{\mathop \prod \nolimits_{1}^{n} {\text{FprobeGAi}}}}}},$$

where ‘FprobeGAi’ refers to the log2(fluorescence level) of a given i probe in gene A. An example of the calculation is given as Supplementary Fig. S4. In this example, for the long non-coding RNA RP11-113I22.1, the strongest splicing index is given for the probe PSR0500147825.hg.1. It appears that the abnormal splicing compared to the rest of the probes occurs clearly for some placentas but not for all.

Library preparation, hybridization and data acquisition were performed by GENOM`IC platform according to manufacturer`s instructions. ClariomD microarray data were extracted using the Transcriptomic Analysis Console provided by Affymetrix. The data from the control subjects of the two cohorts were merged to generate the control group data. When compared against the PE or the IUGR data, a batch correction was applied using the built-in *Batch* parameter in TAC.

Genomic DNA samples were genotyped using the Infinium OmniExpress-24 kit (Illumina) array, in collaboration with Plateforme P3S, Sorbonne Université – site Pitié Salpêtrière, Paris, France. Data were extracted using GenomeStudio 2.0.

### Real time-qPCR

Four µg of RNAs from each placental sample were pre-treated with DNAse-I to remove genomic DNA contamination with RQ1 RNase Free DNase (Promega) according to manufacturer's instructions. The DNAse-I treated RNA samples were then retrotranscribed to cDNA, using the M-MLV kit (Invitrogen) according to manufacturer's instructions. Placental cDNAs were analyzed by RT-qPCR with SensiFAST SYBR No-ROX One-Step Kit (Bioline). To calculate probe expression levels, -ΔCt was calculated by normalizing individual Ct values for each probe of interest, to house-keeping gene (SDHA) Ct. Primers are listed Supplemental Table S7.

### Statistical analyses and computational details

Institut Cochin Cohort full cohort and Angers Control Samples were used for sQTL interrogation. SNPs whose three genotypes (AA, AB, BB) were not represented in at least two samples were discarded (corresponding to Minor Allele Frequency > 20%). A total of 331,204 SNPs was assessed against 48 individual splicing indexes using MatrixeQTL (R package) under the ANOVA model and accounting for gestational age and sample sex as covariates. This means that a full genotypic model was used, surmising independent effects of the three possible genotypes, and not always additive effects of one given allele.

MatrixeQTL generates a matrix correlating gene expression and SNP variants using an accelerated bioinformatics procedure basically dividing the initial matrix in smaller blocks. For cis-sQTL, the distance parameter was fixed to 1 Mb while for trans-sQTL all the genome was interrogated. Only cis-sQTLs and trans-sQTLs with an FDR < 0.05 were used for downstream analyses.

For the surrogate variable analyses (SVA), we used the SVA package available in R (Leek et al. 2012) on R version 3.6.3 (2020–02-29), to estimate latent sources of variations (surrogate variables) in our datasets. We considered three different subsets of samples: (1) CTRL + PEall (PE only + PE_IUGR), (2) CTRL + PEonly, and (3) CTRL + IUGR. The analysis was performed stratified by cases and controls thanks to the inclusion of the disease status variable, which described whether the sample had disease (= 1) or if it was a control sample (= 0). The *mod* and *mod0* parameters in the SVA package allow to build a linear model that describes the gene expression dataset according to the included variables with the function *model matrix.* The two models were built as follow, in this case disease status is the Variable of Interest:
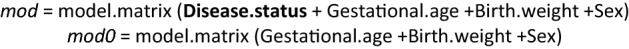


Then the *num.sv* function was used to see whether differences between the two linear models can be explained only by the presence of the variable of interest (disease status) or if any other surrogate variables can be identified, followed by the *sva* function to test whether these surrogate variables are significant:
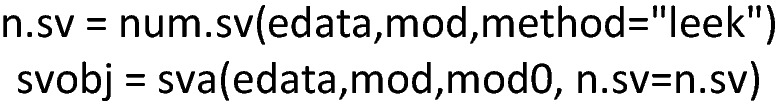


No additional surrogate variables were found to play a role on gene expression differences in any of the three datasets analyzed. At this stage we wanted to identify genes that could be significantly associated with either of the variables: disease status, gestational age, birth weight, Sex. In order to do so the, Variable Of Interest was assigned in turn to each of the variables i.e. disease status, gestational age, birth weight, sex, followed by* p value* function that performs a F-statistic test to assign p-value to each gene in relation to the influence of the Variable of Interest under scrutiny (significance threshold at *p* val < 0.05). For example, to identify genes potentially influenced by “gestational age” the following code was used:
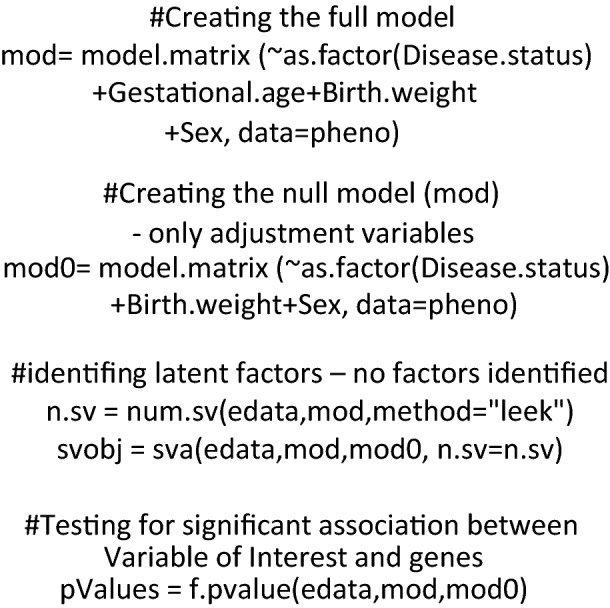


In the boxplots, correlation of Individual Splicing Index (ISI) of the gene with the locus genotype was assessed by one-way ANOVA. Group–group comparison of means was executed by *T* test (*p = 0.05, **p = 0.01, ***p = 0.001, ****p = 0.0001). Linear regression was performed to determine the effect of genotype on ISI in each cis-QTL represented.

Gene enrichment analysis was performed using the Gene Set Enrichment Analysis module available in WebGestalt. The principle of enrichment relative to any database used as reference is the following. The list of ranked genes of the study is compared to a given set of genes specific of a biological function, a gene ontology term, or a given disease, for instance. The rank of occurrence of each gene is recorded and compared to the average expected density (for instance 100 genes out of 10,000 are expected to occur at a frequency of one every 100 genes. When the density is higher than expected, the enrichment score increases. It will reach a maximum or a minimum that will be compared with 1000 random rankings, and the actual value obtained will be compared with the random values yielding a p-value and an enrichment score. The reference databases analyzed are KEGG (https://www.genome.jp/kegg/), Reactome (https://reactome.org/), OMIM (omim.org), GO (http://geneontology.org/).

For RT-qPCR analyses, statistical significance of differences in expression levels of each tested region was assessed by analyzing the -ΔCt distributions between groups (Ctrl (*n* = 13), PE (*n* = 15), PE + IUGR (*n* = 5) and IUGR (*n* = 10)) by 1-way ANOVA followed by Dunnett’s multiple comparison test between diseased groups and control (*p < 0.05; **p < 0.01; ***p < 0.001).

The Monte-Carlo analysis was carried out using a homemade program in VisualBasic accompanying an Excel sheet entitled trans_sQTL_Relative splicing ANOVA, provided as a supplementary material.

The circus plot graph was generated according to the following documentation: https://jokergoo.github.io/circlize_book/book/.

## Discussion

In this study, we present for the first time, to the best of our knowledge, a systematic analysis of alternative splicing alterations in the pathological human placenta. After describing splicing in IUGR and PE compared to control placentas, we validated the finding at the exon level through targeted quantitative RT-qPCR. The splicing index alterations that we observed may be a consequence of the disease, or maybe driving the disease (although in some cases, they can be indirectly triggered by birth weight or shorter gestational age). In fact, the two mechanisms can be operative differently for some specific genes. The large published GWAS analysis of preeclampsia (McGinnis et al. 2017), revealed a SNP in the vicinity of the gene FLT1 (rs4769613), not in the coding region, possibly influencing splicing, that we did not find significant in our study probably due to the limited size of our sample. Indeed, our current sample size qualify our study as exploratory; with a larger sample size, other sQTL with milder effects could be found. In the specific case of sFLT1, one can imagine that a variant may influence splicing and result in being disease-prone. Currently, the cause or consequence question cannot be resolved directly in our study, but is an interesting open question that could be addressed in cell and animal models.

Another interesting question is the relation between placenta eQTL (Peng et al. 2017; Kikas et al. 2019) and the splicing QTL that we identified here. Peng and coworkers surmised that the genes affected in the placenta may mark future risks for the patients (cardiovascular, neurologic). We compared our list of 48 top cisQTL corresponding to 12 different genes with the cis eQTL (3218 cis eQTL were found as associated to SNP variant at the expression level). Four genes were in common between the two lists (33%). A contingency Chi-square calculated between the two datasets revealed a marginally significant enrichment (*p* = 0.034), while none was detected for the trans eQTL. This suggests that there may be a link between expression and splicing regulation in the placenta.

Amongst the genes that we found alternatively spliced in disease, FLT1 was identified amongst the genes that are up-regulated as well as highly differentially spliced in both pathological states, which is a good confirmation of the approach, given the abundant literature positing FLT1 alternative splicing as a hallmark of preeclampsia. The quantitative analysis at the exon level revealed large heterogeneities along the mRNA molecules. We chose primer pairs located in the 1st exon, the 12th exon, the 14th exon, the 28th and between the 15th and the 17th exon. There were significant differences in expression in the first exon that is common to the different isoforms, confirming the well-known induction of this gene in the disease state. The significance weakened for exon 12 and 14, indicating that this increase in expression was untrue for this part of the gene, suggesting a relative decrease of long isoforms encompassing exons 12 and 14 in pathological placentas. When the farthest exon was analyzed (E28), the difference is also significant but to a lesser extent than for Exon 1.The induction ratio in disease compared to control was ~ 11-fold in exon 1, and ~ fivefold in exon 28. This indicates that short isoforms are increased in the pathological state. The data are consistent with an overall induction of the gene in disease, with a more pronounced overexpression of the shorter isoforms.

CLDN1 profile was opposite to FLT1, with a trend towards decrease in pathological state in the 3′ region of the gene. The differential expression does not concern the region located in the 5′ region of the first exon (E1c). However, at the 3′ region of the same exon (E1d), a decreased expression in PE, but not in IUGR is observed. The same trend applies to the two more 3′ regions of exon 2, 3 and 4. The amount of the protein coded by CLDN1 is significantly reduced in PE placentas (Lievano et al. 2006). Here, we show that the two CLDN1 shortest transcripts are differentially expressed in PE, but not the longest one.

Another example of a gene where differentially expressed transcripts can be identified in disease is the one coding for leptin (LEP). As expected, LEP was highly upregulated at the gene level in disease compared to control, confirming numerous previous observations, including from our group (Madeleneau et al. 2015; Biesiada et al. 2019). The alternative splicing profile of LEP showed that the first part of the gene (exons 1–2a) has a higher expression in diseased placentas as compared to controls. We designed primers to interrogate the expression of exon 2a, found only in three out of the four isoforms putatively expressed in the tissue (LEP-201, LEP—Affx TR0700064863.hg, LEP—AffxTR0700064865.hg), exon 2b common to all isoforms, and finally of the boundaries between Exons 3b and 3c (encompassed in LEP-201 and LEP– Affx TR0700064863.hg). The RT-qPCR results showed a significant upregulation of all three tested regions in PE compared to control, whilst only the 2a-2b region showed a higher expression in IUGR samples. Our results clearly indicate that IUGR and PE have different repertoires of LEP isoforms.

FSTL3 is also known as increased in preeclampsia (Founds et al. 2009; Gormley 2017). In this case, the increase of the gene was observable all along the molecule, but only significantly in 5′ exons. In summary, the middle region of the gene is not deregulated while there is an increase in isoforms were this section could be spliced out. We also present the profile of TXK kinase. While the 5′ part of the gene was not different according to the microarray data, the 3′ appeared selectively deregulated in PE with or without IUGR. This deregulation was confirmed by RT-qPCR as shown by analysis of exons 13 and 14–15 that behave very similarly. This tyrosine kinase has not previously been associated with preeclampsia in the literature, but the mRNA is highly expressed in the placenta (trophoblast and endothelial cells), and the protein participates in immune regulation.

Pathology-driven alterations of alternative splicing require specific mechanisms and a specific molecular machinery, incorporating in particular small nuclear ribonucleoproteins, such as U1, U2, U4, U5 and U6, and spliceosome proteins, as nicely reviewed in (Scotti and Swanson 2016). To date, alternative splicing in complex diseases has essentially been considered in neurological and neuromuscular disorders, such as the abnormal splicing of Tau proteins encoding genes responsible for tauopathies (Bartsch et al. 2016). A paradigmatic example of alternative splicing in disease is the LaminA gene (*LMNA*), whose different dominant mutations induce respectively, and independently, muscular dystrophy, progeria, or dilated cardiomyopathy. In these cases, a monogenic variant induces a complex disease. However, understanding altered splicing in the context of polygenic and highly multifactorial diseases and processes is trickier, but starts to be addressed. For instance, in the aging brain, alternative splicing was recently studied in 44 mouse brain areas (Li et al. 2019). Interestingly, premature aging of the placenta is thought as a putative general feature of preeclampsia (Manna et al. 2019).

Any evaluation of the splicing species for complex diseases or conditions requires an accurate description of the spliced species found in the relevant organ. In obstetric diseases, this pivotal organ is the placenta. Therefore, a first effort should be carried out to evaluate the diseased placenta, which is the exact purpose of the present article. Here, we demonstrate that genes that harbor differential splicing are indeed a component in the pathogenesis of PE and IUGR. These associations with splicing are not systematically associated with a difference in the overall level change of a given mRNA, and are partly piloted by genomic variants.

Our sample size is relatively small, and our results have to be considered as a proof-of-concept warranting other targeted studies. Nevertheless, the simulation data presented in (Huang et al. 2018), indicate that a QTL with an effect of > 1 Standard Deviation is detected with sample of the size of ours, if the Minor Allele Frequency is above 0.2 (which is the case here, since we selected SNPs for which each genotype was carried by at least two individual samples}. Also for individual probes, we have a QTL effect > 1 SD in many cases, due to our stringent selection of the largest splicing indexes. Independently of variations in the expression level, we found that the subset of genes that are alternatively spliced have a disease-specific profile, with marked differences between preeclampsia and IUGR, showing in particular that the first condition but not the second, correlates with brain diseases. This fascinating observation is in accordance with many recent studies showing strong links between preeclampsia and brain vascular diseases (Andolf et al. 2020; Patel et al. 2020; Miller 2019; Basit et al. 2018; Cheng and Sharma 2016). For example, deregulation of alternative splicing of the gene STOX1, involved in preeclampsia and Alzheimer's disease (Dijk et al. 2005,2010) induces a disequilibrium of its two isoforms and increases the risk of disease (Vaiman and Miralles 2016; Ducat 2020).

Focusing our work on 48 genes that were found strongly alternatively spliced between healthy samples and disease, we showed that genetic variants (SNP) impact these splicing differences. On the one hand, the cis-sQTLs had an additive effect, which is consistent with an impact of the genetic variant either on the mRNA tridimensional structure, or on the binding of splicing factors, that would affect the splicing of the RNA molecule carrying the variant. On the other hand, trans-sQTLs could give insights on how specific loci act on splicing at various genome locations. After a stringent screen, we show an allele-specific impact of four trans sQTL loci on splicing.

The influence of the cis and trans-sQTL on splicing was generally disconnected from the disease state. This issue is not surprising since, albeit we initially restrict our genes of interest to genes that are alternatively spliced in disease, this screen was preferably used to detect genes that are highly splicing-prone. The genetic determination of the splicing is driven by specific sQTLs that may not be a strong determinant of the disease status, compared to other regulators modulating, for instance, gene expression levels. One exception could be rs1431647. This SNP located in the long noncoding RNA LOC105375897 is systematically associated with the disease when the SNP is heterozygous, associated systematically in this case to divergent splicing for FLT1, FSTL3, P4HA1, NTRK2, FLT4, BAIAP2, CLDN1 and ST18. The ways these variants play their function remains an open mechanistic question.

Splicing in the placenta was observed previously for collagen 1 (Type XIII collagen) in the early human placenta (Juvonen et al. 1993). A more systematic approach was used by Kim and coworkers (Miyagawa et al. 2012), revealing that compared to other tissues, the splicing in the placenta presents specific aspects; interestingly, they found that enrichment of genes involved in placental anomalies and pre-term birth are selectively enriched in the set of placental alternatively spliced genes. One of the most studied gene in terms of pathological alternative splicing is FLT1, recently shown to be alternatively spliced through the action of U2AF65 and JMJD6 (Eddy et al. 2020).

In summary, these analyses de novo re-identified a known factor of preeclampsia and IUGR, alternative splicing of FLT1, which validates the approach, and found hundreds of other disease-associated genes that are affected by splicing. We also show that these alterations are potentially genetically based. This present study paves the way towards a systematic exploration of the mechanisms and consequences of alternative splicing in normal and pathological placental function.

## Supplementary Information

Below is the link to the electronic supplementary material.

Supplementary Figure S1: A network of genes alternatively spliced in PE and IUGR (generated using String- https://string-db.org/). The major molecular functions involved in this common network are based on binding molecules of the extracellular matrix in particular. Supplementary Figure S2: Analyses of alternative splicing events by targeted qRT-PCR. The upper part of the graph for each gene are output from the TAC software. The intermediate graph represents the position of the various primers used and the lower part the actual results (*p < 0.05, ** p<0.01 and ***p < 0.001). Supplementary Figure S3: Evaluation of the batch effect and other additional putative variables by Eigen correlation analysis. The upper graph show the PCA distribution of the samples according to the disease status. In the middle graph, the same plot is shown with the origin of the samples (Cochin and Angers). A third labeling with the sex distribution is presented. The lower panel presents the correlation of the different variables with the PCA axes. The first axis (31.88% of the variance correlates with Disease group, Weight, and Gestational age (GA) and sex is significantly correlated with axes 2 and 3. Ethnicity is associated with PC3. (PC = Principal Component, *p < 0.05, **p < 0.01 and ***p < 0.001). Supplementary Figure S4: This figure presents the way the individual splicing index (ISI) necessary for the sQTL analysis is performed on an example showing the different placentas in columns, the different probes analyzed and the relative splicing index for each placenta calculated as described in the methods. Supplementary Figure S5: Deregulation of gene expression in the non-coding RNA with SNPs associated to trans sQTLs, showing a specific deregulation of LOC105375897 (**p < 0.01) (PPTX 2817 KB)

Supplementary file2 (XLSX 1394 KB)

Supplementary file3 (XLSM 40467 KB)

## Data Availability

The datasets supporting the conclusions of this article are available in the EMBL-EBI repository, E-MTAB-9416.

## Code availability

Monte-Carlo program associated to an Excel file provided as supplementary material).
