# Supplementary material for: Alternative splicing in normal and pathological human placentas is correlated to genetic variants
Source: Hum Genet. 2021 Jan 12;140(5):827–48. doi: 10.1007/s00439-020-02248-x (PMC8052246; doi:10.1007/s00439-020-02248-x)
Supplement: Supplementary file 1 — Supplementary Figure S1: A network of genes alternatively spliced in PE and IUGR (generated using String- https://string-db.org/). The major molecular functions involved in this common network are based on binding molecules of the extracellular matrix in particular. Supplementary Figure S2: Analyses of alternative splicing events by targeted qRT-PCR. The upper part of the graph for each gene are output from the TAC software. The intermediate graph represents the position of the various primers used and the lower part the actual results (*p < 0.05, ** p<0.01 and ***p < 0.001). Supplementary Figure S3: Evaluation of the batch effect and other additional putative variables by Eigen correlation analysis. The upper graph show the PCA distribution of the samples according to the disease status. In the middle graph, the same plot is shown with the origin of the samples (Cochin and Angers). A third labeling with the sex distribution is presented. The lower panel presents the correlation of the different variables with the PCA axes. The first axis (31.88% of the variance correlates with Disease group, Weight, and Gestational age (GA) and sex is significantly correlated with axes 2 and 3. Ethnicity is associated with PC3. (PC = Principal Component, *p < 0.05, **p < 0.01 and ***p < 0.001). Supplementary Figure S4: This figure presents the way the individual splicing index (ISI) necessary for the sQTL analysis is performed on an example showing the different placentas in columns, the different probes analyzed and the relative splicing index for each placenta calculated as described in the methods. Supplementary Figure S5: Deregulation of gene expression in the non-coding RNA with SNPs associated to trans sQTLs, showing a specific deregulation of LOC105375897 (**p < 0.01) (PPTX 2817 KB) [file 439_2020_2248_MOESM1_ESM.pptx]

## Slide 1
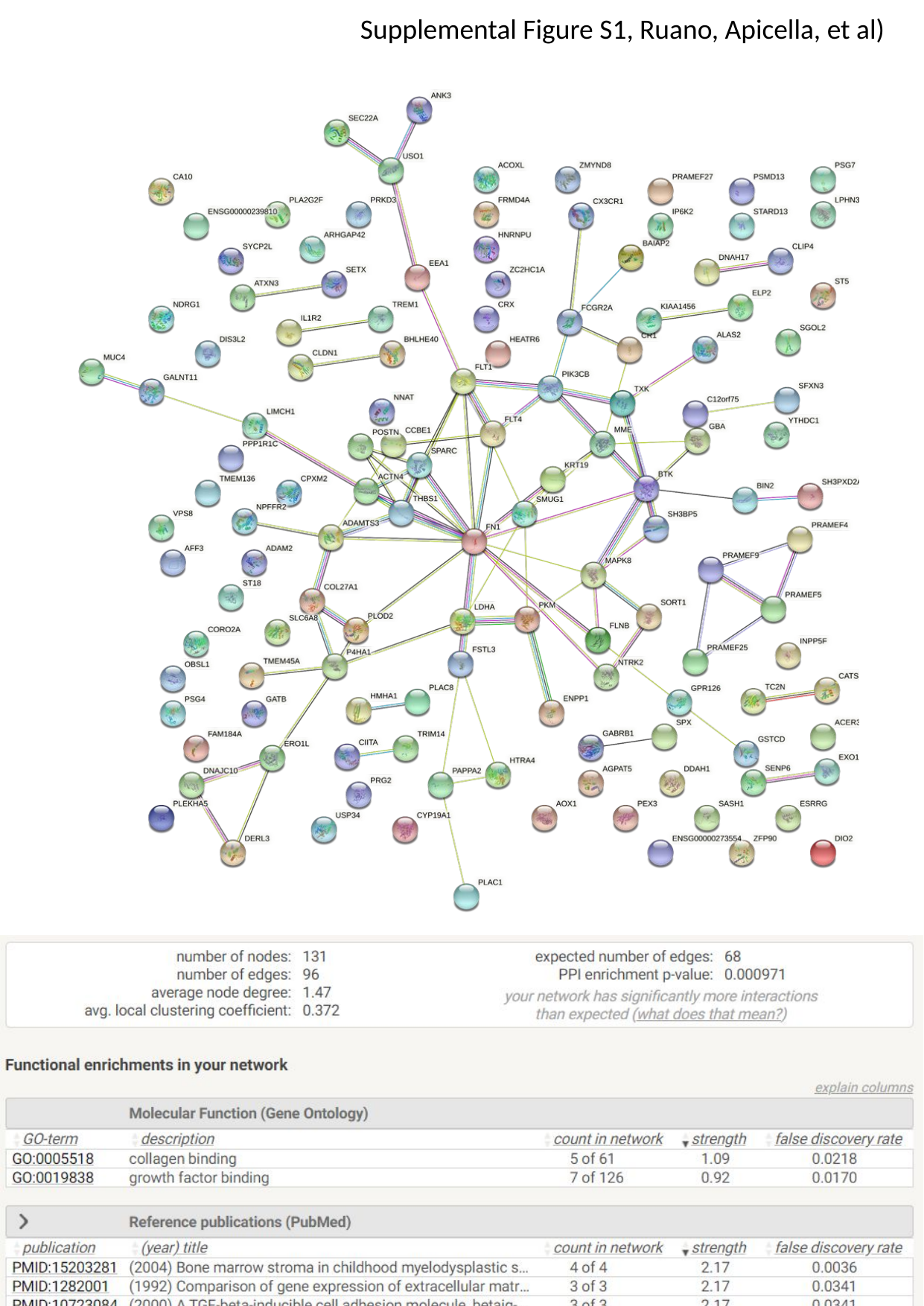

Supplemental Figure S1, Ruano, Apicella, et al)

## Slide 2
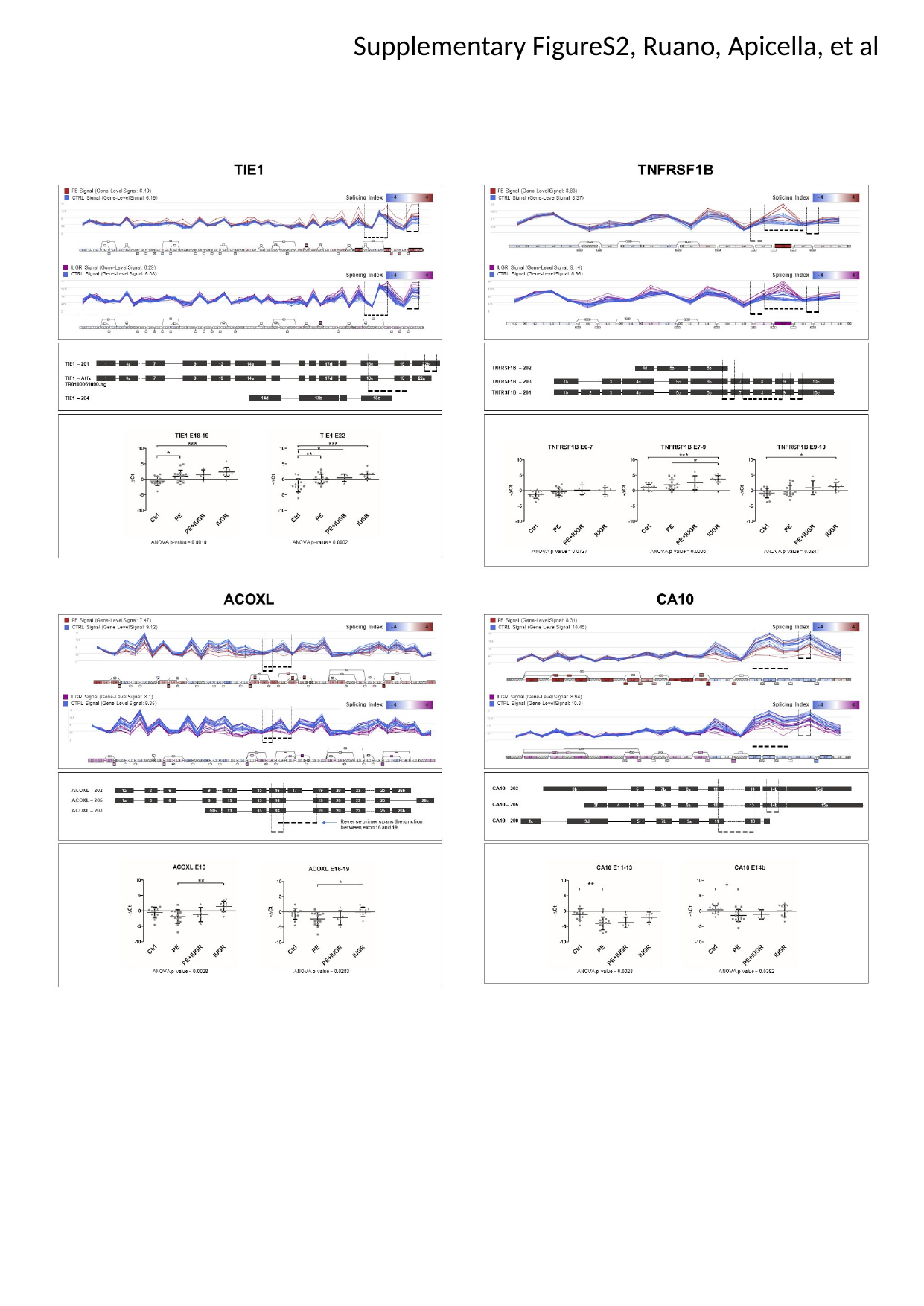

Supplementary FigureS2, Ruano, Apicella, et al

## Slide 3
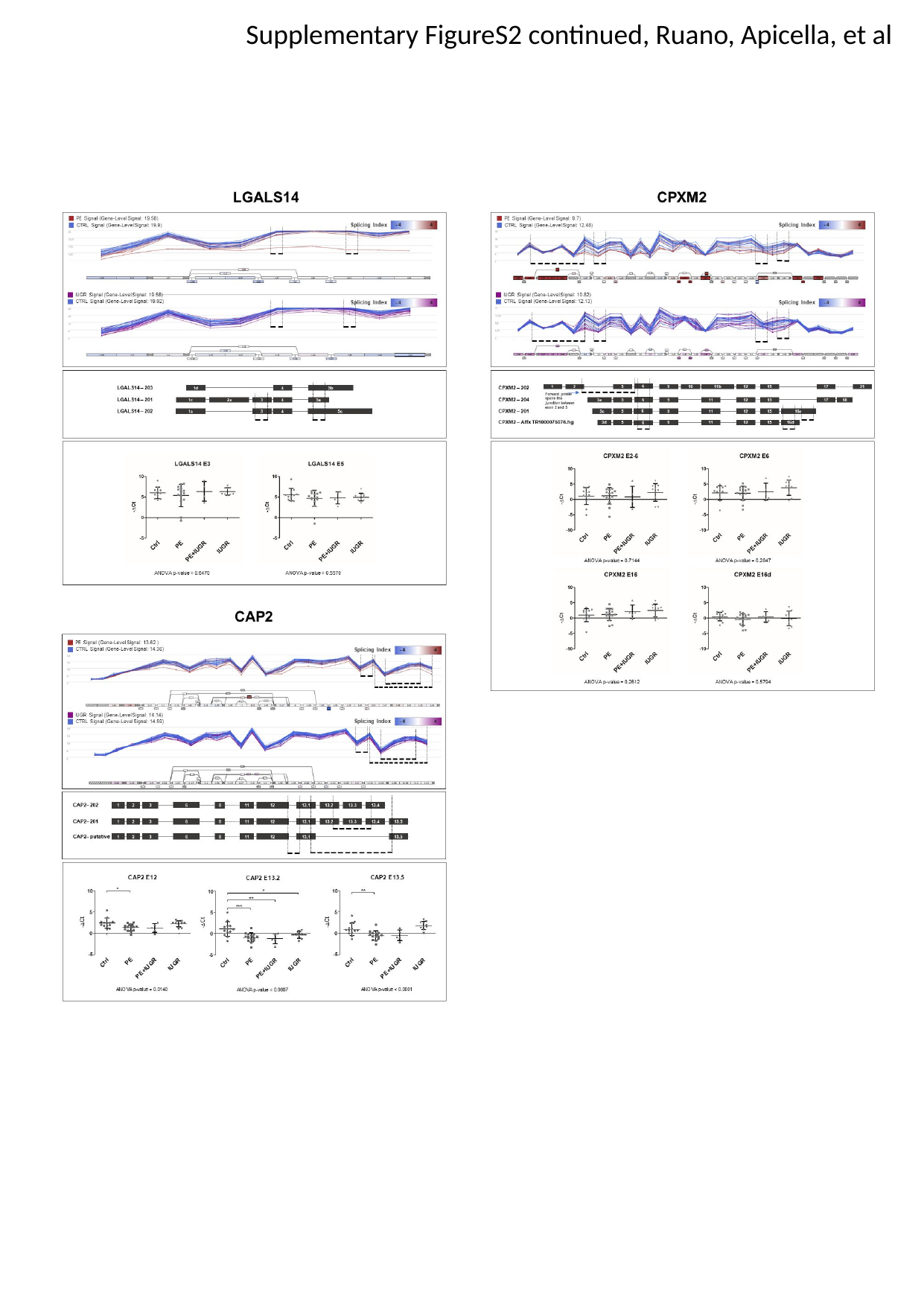

Supplementary FigureS2 continued, Ruano, Apicella, et al

## Slide 4
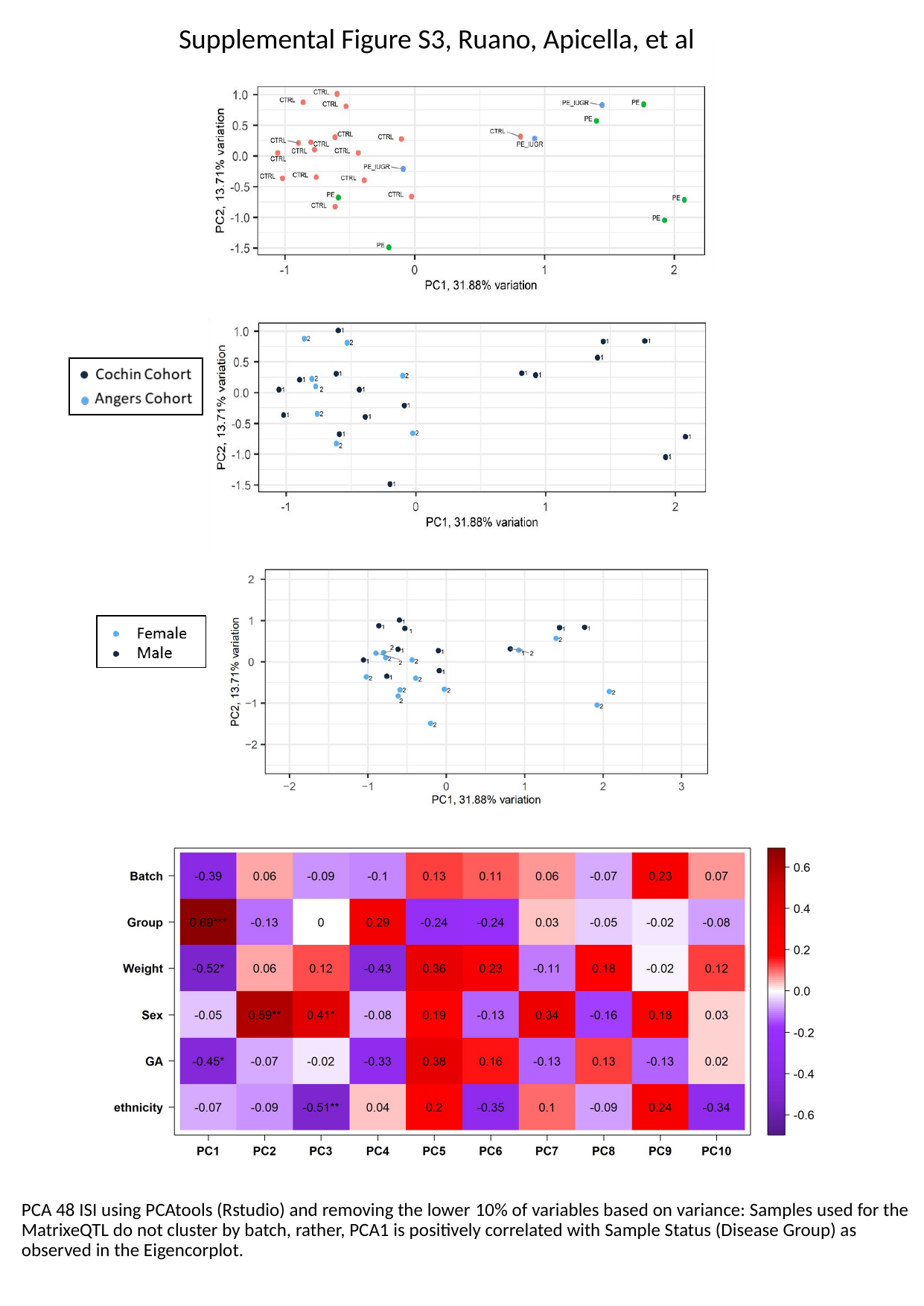

Supplemental Figure S3, Ruano, Apicella, et al
PCA 48 ISI using PCAtools (Rstudio) and removing the lower 10% of variables based on variance: Samples used for the MatrixeQTL do not cluster by batch, rather, PCA1 is positively correlated with Sample Status (Disease Group) as observed in the Eigencorplot.

## Slide 5
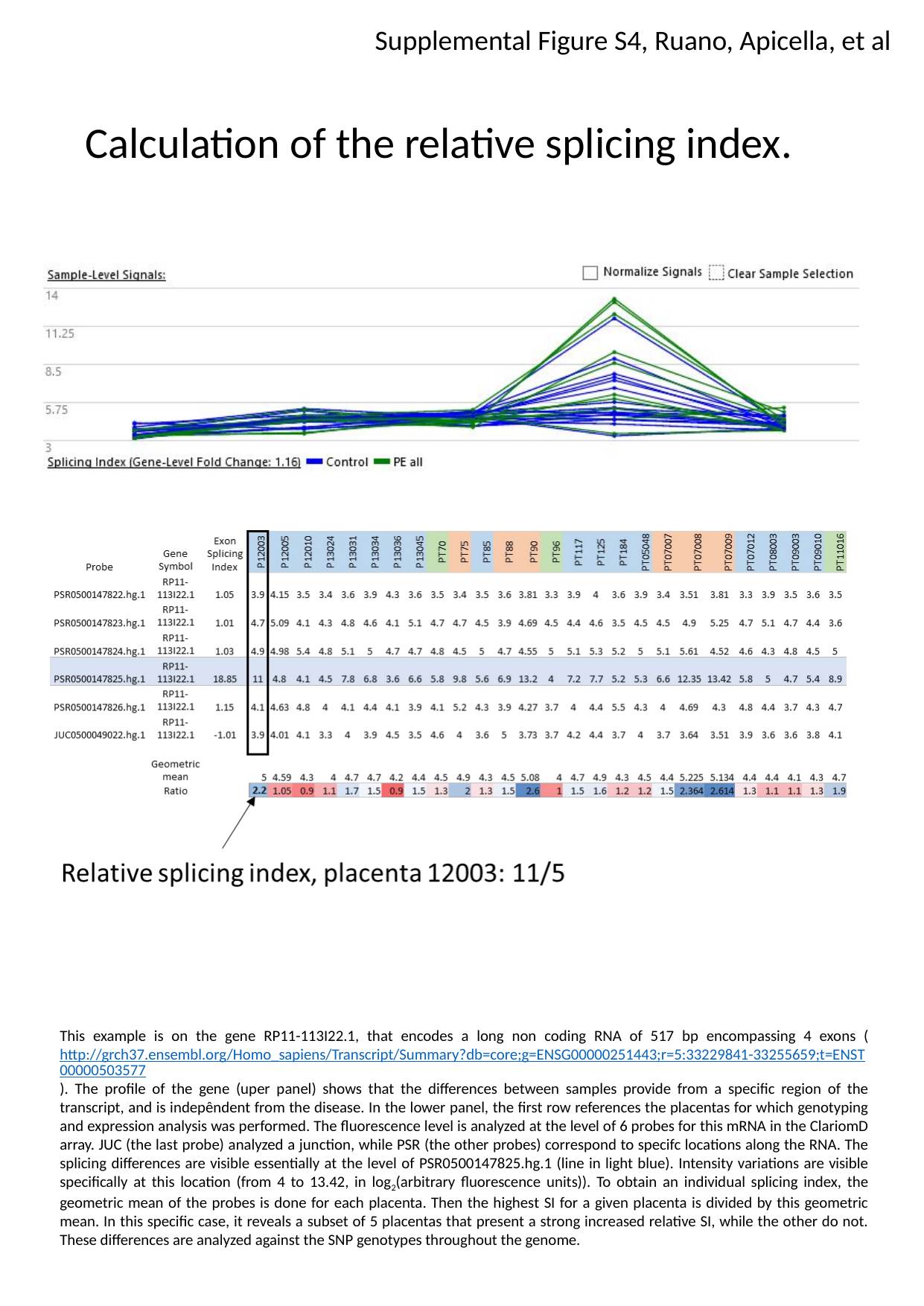

Supplemental Figure S4, Ruano, Apicella, et al
Calculation of the relative splicing index.
This example is on the gene RP11-113I22.1, that encodes a long non coding RNA of 517 bp encompassing 4 exons (http://grch37.ensembl.org/Homo_sapiens/Transcript/Summary?db=core;g=ENSG00000251443;r=5:33229841-33255659;t=ENST00000503577). The profile of the gene (uper panel) shows that the differences between samples provide from a specific region of the transcript, and is indepêndent from the disease. In the lower panel, the first row references the placentas for which genotyping and expression analysis was performed. The fluorescence level is analyzed at the level of 6 probes for this mRNA in the ClariomD array. JUC (the last probe) analyzed a junction, while PSR (the other probes) correspond to specifc locations along the RNA. The splicing differences are visible essentially at the level of PSR0500147825.hg.1 (line in light blue). Intensity variations are visible specifically at this location (from 4 to 13.42, in log2(arbitrary fluorescence units)). To obtain an individual splicing index, the geometric mean of the probes is done for each placenta. Then the highest SI for a given placenta is divided by this geometric mean. In this specific case, it reveals a subset of 5 placentas that present a strong increased relative SI, while the other do not. These differences are analyzed against the SNP genotypes throughout the genome.

## Slide 6
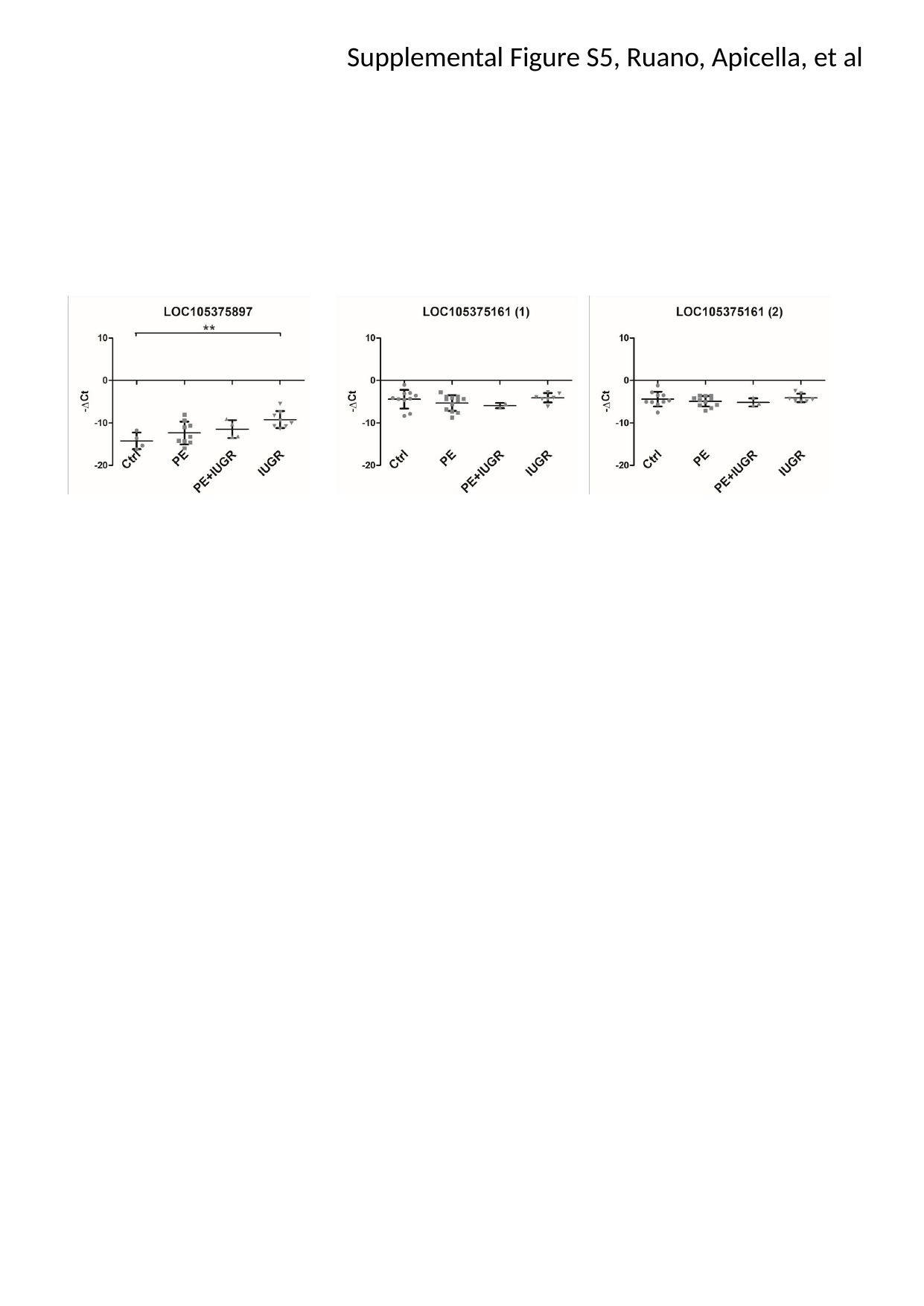

Supplemental Figure S5, Ruano, Apicella, et al
